# Semisynthetic Amides of Amphotericin B and Nystatin A_1_: A Comparative Study of In Vitro Activity/Toxicity Ratio in Relation to Selectivity to Ergosterol Membranes

**DOI:** 10.3390/antibiotics12010151

**Published:** 2023-01-11

**Authors:** Anna Tevyashova, Svetlana Efimova, Alexander Alexandrov, Olga Omelchuk, Eslam Ghazy, Elena Bychkova, Georgy Zatonsky, Natalia Grammatikova, Lyubov Dezhenkova, Svetlana Solovieva, Olga Ostroumova, Andrey Shchekotikhin

**Affiliations:** 1 Gause Institute of New Antibiotics, 11 B. Pirogovskaya, 119021 Moscow, Russia; 2 Institute of Cytology of the Russian Academy of Sciences, 4 Tikhoretsky, 194064 St. Petersburg, Russia; 3 Federal Research Center “Fundamentals of Biotechnology” of the Russian Academy of Sciences, Bach Institute of Biochemistry, 33 Leninsky Ave., bld. 2, 119071 Moscow, Russia; 4 Institute of Biochemical Technology and Nanotechnology, Peoples’ Friendship University of Russia (RUDN), 6 Miklukho-Maklaya Street, 117198 Moscow, Russia; 5 Department of Microbiology, Faculty of Pharmacy, Tanta University, Tanta 31111, Egypt

**Keywords:** polyene antibiotics, amphotericin b, nystatin, semisynthetic derivatives, amidation, water-soluble, antifungal activity, ergosterol, membrane permeabilization, cytotoxicity, hemolysis

## Abstract

Polyene antifungal amphotericin B (AmB) has been used for over 60 years, and remains a valuable clinical treatment for systemic mycoses, due to its broad antifungal activity and low rate of emerging resistance. There is no consensus on how exactly it kills fungal cells but it is certain that AmB and the closely-related nystatin (Nys) can form pores in membranes and have a higher affinity towards ergosterol than cholesterol. Notably, the high nephro- and hemolytic toxicity of polyenes and their low solubility in water have led to efforts to improve their properties. We present the synthesis of new amphotericin and nystatin amides and a comparative study of the effects of identical modifications of AmB and Nys on the relationship between their structure and properties. Generally, increases in the activity/toxicity ratio were in good agreement with increasing ratios of selective permeabilization of ergosterol- vs. cholesterol-containing membranes. We also show that the introduced modifications had an effect on the sensitivity of mutant yeast strains with alterations in ergosterol biosynthesis to the studied polyenes, suggesting a varying affinity towards intermediate ergosterol precursors. Three new water-soluble nystatin derivatives showed a prominent improvement in safety and were selected as promising candidates for drug development.

## 1. Introduction

**Figure 1:**
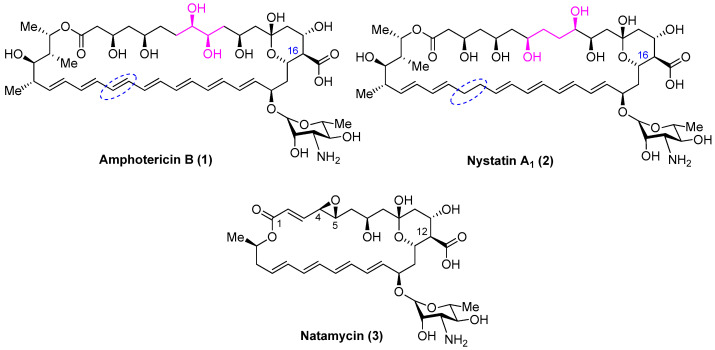
Structure of polyene antibiotics amphotericin B, nystatin A_1_ and natamycin.

Systemic fungal infections, which are a common comorbidity in immunocompromised patients, are characterized by severe symptoms and high mortality rates [1,2]. The first-line antifungals used for chemotherapy of invasive mycoses include azoles and echinocandins; however, their long-term use results in the widespread emergence of resistant pathogenic fungi [3]. The coronavirus pandemic, COVID-19, announced in 2020, led to a dramatic increase in the prescription of antibiotics to treat or prevent the development of bacterial and fungal complications in immunocompromised patients [4]. As a result, a significant increase in antimicrobial resistance both to antibacterial and antifungal agents is being reported [5,6,7]. Thus, polyene macrolide amphotericin B (Figure 1, AmB, **1**), which has a low rate of resistance emergence, is still the most widely used antifungal in intensive care, though its use is limited by nephrotoxicity and very low water solubility [8]. Amphotericin B is also the drug of choice for mucormycosis treatment [9]. This condition has become more frequent, mostly due to patients hospitalized with coronavirus infections [10].

The other significant polyene macrolides in clinical use are nystatin A_1_ (Figure 1, Nys, **2**) and natamycin (Figure 1, Nat, **3**) (Figure 1); however, these are not used systemically. Common structural features shared by AmB, nystatin and natamycin include a conjugated double-bond system and the carbohydrate residue mycosamine.

Research on the mechanism of action of polyene antibiotics has been conducted since the 1970s and remains relevant nowadays [11]. Initial studies in this area revealed that AmB causes membrane permeabilization via interaction with sterols [12,13,14,15] and, in 1974, the barrel-stave model was proposed [16]. For decades, pore-formation activity was considered the main mode of the fungicidal activity of AmB and Nys, and the higher affinity of polyenes to ergosterol (ERG) vs. cholesterol (CHOL) was considered to be the likely explanation for their selectivity against fungal cells [17,18,19]. As for Nat, a smaller member of the polyene antifungal family, widely used in medicine and agriculture, the mode of action has remained unclear up to 2008, when Y.M. te Welscher et al. found that Nat did not permeabilize membranes, and its antifungal action was realized through binding to ergosterol [20]. Subsequent investigations showed that natamycin, via its interaction with ergosterol, inhibits amino acid and glucose transport across the plasma membrane [21]; moreover, other polyenes (AmB and Nys) are thought to share this mode of action, which is masked by their ability to form pores. In recent years, M. Burke’s group has claimed that pore formation is not necessary for the antifungal action of AmB [22] and that it kills fungal cells by forming extramembranous aggregates, thereby extracting ergosterol from lipid bilayers [23]. In 2021, they expanded this sterol-sponge mode of antifungal action to other glycosylated polyene macrolides [24]. According to this model, the separation of the pore-forming activity and the ability to extract ergosterol through the modification of polyene’s structure is a promising way to obtain less toxic antifungals [25]. However, there are contrary opinions about this theory. Delhom et al. proved the ability of AmB to extract ERG from lipid bilayers, but this was not observed for CHOL-containing bilayers. This observation partly supports the sterol-sponge model [26]. Daniel M. Kaminski cast doubt on the relevance of this model in living organisms [27]. Yamamoto et al. demonstrated that AmB molecules are located predominantly parallel to the lipid bilayer [28], which supports the barrel stave model. Subsequent work resolved the entire structure of the AmB channel assembly in ergosterol-containing membranes by NMR, as well as molecular dynamics calculations, thus claiming that “other possible structures, such as the sterol sponge model, are considered highly unlikely” [29]. Among the secondary effects of AmB, the most prominent is the induction of oxidative stress, leading to fungal cell death [30,31].

Due to their amphiphilic structure, polyenes tend to form aggregates, and the influence of aggregation on activity/toxicity ratio for AmB has been investigated. AmB can permeabilize ERG-containing phosphatidylcholine membranes in a monomeric state as well as in an aggregated form; while in the case of CHOL-containing and sterol-free membranes, AmB in its monomeric form lacks pore-forming activity [32]. Furthermore, monomeric AmB solutions are less toxic than the solutions of Fungizone, a form in which the polyene is in almost completely self-aggregated form [33]. Finally, a series of papers [34,35,36] presented less toxic semisynthetic derivatives of AmB with decreased dimerization in water solutions due to a lack of zwitterionic properties. This supports the idea that the reduction of the dimerization properties of AmB is a promising approach to obtain less toxic antifungal agents.

The aim of our research was to create new, less toxic semisynthetic polyenes with increased solubility in water, suitable for the further development of next generation antifungal drugs for the treatment of systemic mycoses. The presented data shows that two factors mainly lead to a decrease in toxicity: an increase in specific affinity to ergosterol and a decrease in the tendency to aggregate.

Thus, we present a new series of nystatin amides and compare these with new and previously published amides of AmB in terms of their activity/toxicity ratio in vitro on cells and on model Erg- and Chol-containing membranes. We decided to focus on the amidation of the carboxylic group, because this modification is both simple to introduce and has a high impact on the activity/toxicity ratio, as first reported in 1989 [37] and later supported by a series of papers [34,35,38,39,40], describing safer and more highly active water-soluble polyene amides, which in most cases, contain an additional basic residue. We also obtained indications that polyene amides may differ in their affinity to various ergosterol precursors, which is relevant to drug-resistant fungal strains.

## 2. Results

### 2.1. Synthesis of Polyene Amides

**Scheme 1:**
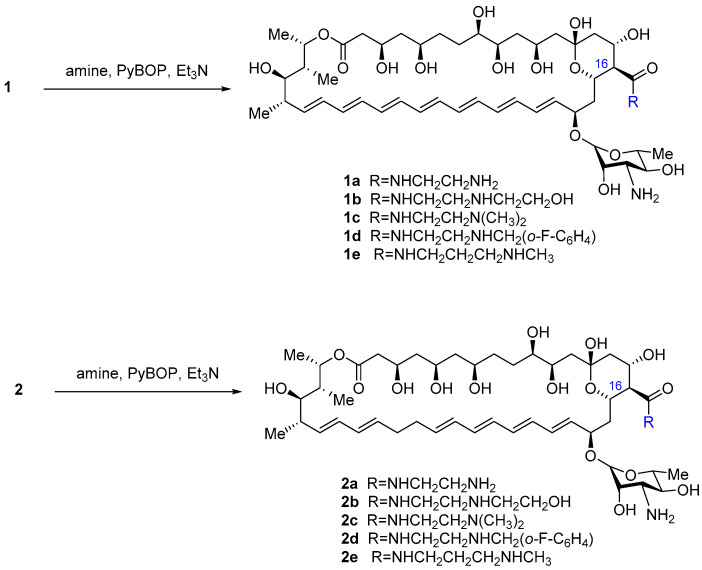
Synthesis of polyene antibiotic amides.

Chemical modification involved the direct amidation of the C16-carboxylic group of the antibiotic by the corresponding diamine in the presence of benzotriazol-1-yl-oxytripyrrolidinophosphonium hexafluorophosphate (PyBOP) and the subsequent purification of the obtained carboxamide by column chromatography (Scheme 1).

The yields of the target amides **1c**–**1e**, **2a**–**2e** varied from moderate to good (20–60%) due to the well-known lability of polyene antibiotics, their sensitivity to light and oxygen, and basic/acid conditions. AmB derivatives **1a**,**b** were described earlier [35]. All the new amides had considerably higher solubility in water compared to their parent antibiotics. For several of them, the determination of the solubility was performed using UV spectroscopy of standard and saturated solutions according to the method described previously [41,42] with minor modifications. Saturation of the solutions at a concentration of 100 g/L was not achieved for the compounds **1a**,**c**,**e**, and **2a**,**c**,**e**, thus indicating a solubility of more than 100 g/L (solubility of AmB–0.75 g/L, Nys–0.36 g/L [43]).

The purity of new amides **1a**–**1e**, **2a**–**2e** was determined by TLC and HPLC, and the structure was confirmed by HR-ESI mass-spectrometry and NMR spectra. To fully assign signals in both the ^1^H and ^13^C spectra, a set of 2D experiments was performed: ^1^H-^1^H COSY, TOCSY, NOESY (ROESY), HSQC, HMBC, and H2BC. The latter experiment was especially useful for assigning resonances from the polyene part of the molecules. In addition, a set of selective TOCSY experiments was performed in order to assign the signals of the H-19, H-20, H-33, and H-34 atoms of the polyene part. The assignment of ^1^H and ^13^C signals for compounds **1c**–**1e**, **2a**–**2e** is shown in Table S1 (Supporting information).

### 2.2. In Vitro Antifungal Activity and Toxicity toward Mammalian Cells

The antifungal activity of polyene derivatives **1a**–**1e**, **2a**–**2e** was tested against strains of *Candida spp.* (reference strains *C. parapsilosis* ATCC 22019, *C. albicans* ATCC 10231 and clinical isolates *C. krusei* 432M, *C. albicans* 604M, *C. albicans* 8R, *C. glabrata* 61L, *C. tropicalis* 3010, *C. parapsilosis* 58L) and filamentous fungi (*Aspergillus fumigatus* ATCC 46645, *Aspergillus niger* 37a and *Trichophyton rubrum* 2002) and compared to that of AmB (**1**) and Nys (**2**) using the broth microdilution method, as described in the EUCAST definitive documents [44,45] (Table 1). Reference strain *C. parapsilosis* ATCC 22019 was used as the control in each experiment. In all experiments, the in vitro microdilution technique in 96-microwell plates was applied. The minimum inhibitory concentration (MIC) was defined as the lowest concentration that resulted in complete growth inhibition after incubation for 24 and 48 h for *Candida* spp. and 48–72 h for dermatophytes.

All of the new Nys derivatives showed good antifungal activity, close to that of parent antibiotic (with the exception of *C. krusei* 432M); in the AmB group, the most prominent results were obtained for amides with ethylenediamine and N-(2-hydroxyethyl)ethylenediamine moieties, **1a** and **1b**, respectively, published earlier.

**Figure 2:**
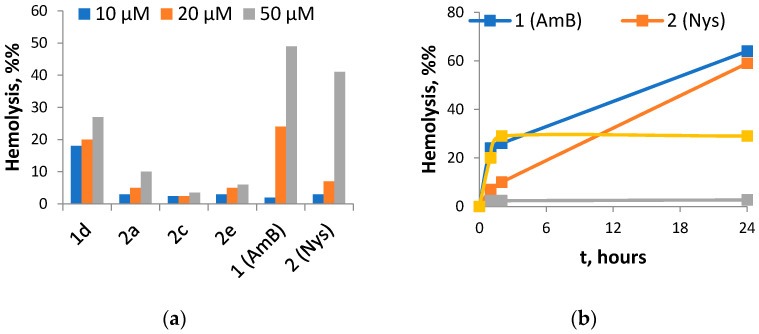
Hemolytic activity of selected amides of Nys and AmB: (**a**) hemolysis ratio after 1 h incubation of red blood cells with 10, 20 and 50 μM of tested compounds; (**b**) hemolysis ratio during 24 h incubation of red blood cells with 20 μM of tested compounds.

A preliminary estimation of the toxicity of the new polyene amides was performed using the MTT test on human embryonic kidney cells (HEK293) (Table 2). The reference drugs used were AmB (**1**), Nys (**2**), and the antitumor antibiotic doxorubicin. For the new compounds with the highest activity/toxicity ratios, we also determined the hemolytic activity (Figure 2).

The most active Nys derivative **2a** was 3-fold less toxic against kidney cells and showed similar hemolytic activity at 10 and 20 μM with notably lower activity at 50 μM, as compared to Nys. Derivatives **2c** and **2e** did not display toxicity against kidney cells and possessed weak hemolytic activity up to 50 μM. After 24 h, the hemolysis ratio of **2c** did not exceed 5%, while Nys demonstrated a ratio of 59%. AmB derivatives were quite toxic to HEK293, and the least toxic derivative **1d** caused rapid hemolysis at all the tested concentrations up to 20–30%, but after 24 h the hemolysis ratio did not change, while native antibiotics (Nys and AmB) showed a gradual increase in membrane permeabilization with time up to 60%.

### 2.3. Electrophysiological Experiments

**Figure 3:**
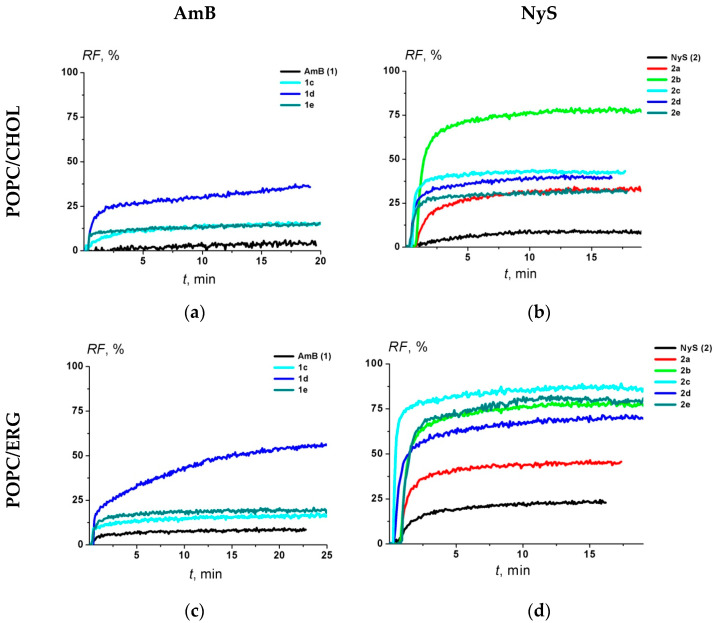
The dependence of the polyene-induced relative fluorescence of calcein (RF, %) leaked from POPC/CHOL (67/33 mol %) (upper panel) and POPC/ERG (67/33 mol %) (lower panel) vesicles on time. The moment of addition of 5 μM of AmB (**a**,**c**) and NyS (**b**,**d**) derivatives into liposomal suspension is referred to as zero point. The relation between the color symbol and the compound is given on the figure.

Figure 3 demonstrates the ability of the parent polyene macrolide antibiotics and their derivatives at 5 µM to disengage calcein from large unilamellar vesicles prepared from 67 mol% palmitoyloleoylphosphocholine (POPC) and 33 mol% CHOL (Figure 3a,b) or ERG (Figure 3c,d). POPC is a hybrid monounsaturated lipid that is made up of one fully saturated 16C chain and one monounsaturated 18C (D9-Cis) fatty acid chain, a variant of phosphatidylcholine, the major abundant phospholipid of eukaryotic cell membranes. The first lipid mixture (POPC/CHOL) imitates the antibiotic action on mammalian cell membranes and the second one (POPC/ERG) mimics fungal membranes. The two-exponential dependences were used to fit the time dependences of calcein release induced by different polyenes (AmB and Nys) and their derivatives from POPC/CHOL- and POPC/ERG-liposomes as a first-order approximation. The characteristic parameters of dependences, the maximal leakage, RF_max_, and the times related to fast and slow components, t_1_ and t_2_, respectively, are presented in Table 3.

The efficacy of the tested AmB derivatives (**1d**, **1c** and **1e**) to disengage calcein from POPC/CHOL vesicles decreased in the following order: **1d** (RF_max_ is about 40%) > **1c** ≈ **1e** (about 15%) > AmB (**1**) (about 6%) (Figure 3a and Table 3). In the case of the POPC/ERG liposomes, the RF_max_ decreased in the same order, but the effects were 1.5-times greater for AmB, **1d**, **1c** and **1e** (Figure 3c and Table 3). In the case of Nys derivatives in the POPC/CHOL vesicles, the RF_max_ decreased in the following order: **2b** (about 70%) > **2c** ≈ **2d** ≈ **2a** (about 40%) > **2e** (about 20%) > Nys (**2**) (about 10%) and in POPC/ERG liposomes it decreased in the series: **2c** ≈ **2b** ≈ **2d** ≈ **2e** (about 80%) > **2a** (about 45%) > Nys (about 20%) (Figure 3b,d and Table 3).

Additionally, we estimated the efficiency index, EI, as a ratio of the maximal leakage of calcein from unilamellar vesicles composed of POPC/ERG (67/33 mol %) and POPC/CHOL (67/33 mol %) (Table 3). The higher EI corresponds to higher safety. It was found that this parameter decreased in the following orders for AmB and NyS derivatives: **1d** ≈ AmB ≥ **1e** ≈ **1c** and **2e** > **2c** ≥ Nys ≈ **2d** > **2b** = **2a**.

As was mentioned above, different polyene macrolides were shown to have different modes of action with the same target—the fungal cell membrane. It is believed that AmB and Nys form pores via interaction with sterols (especially ERG) in the membrane, which then leads to uncontrolled leakage of the intracellular contents and cell death [46].

Diphytanoyl phospholipids, and especially, diphytanoylphosphocholine (DPhPC), are commonly used as model membranes when conducting electrophysiological experiments. DPhPC has two fully saturated 16C fatty acid chains with four methyl groups attached to each chain. It is believed that synthetic phytanyl-chained glycolipid bilayers are promising materials for the reconstitution and transport studies of membrane proteins [47]. This relates to their unique properties, such as high chemical/physical stability (including under mechanical stress), low water permeability, and no gel-to-fluid phase transition at ambient temperature [48]. The high chemical and mechanical stability of phytanoyl lipids is attributed to the entirely saturated alkyl chains, which are much less sensitive to chemical degradation by air or light than unsaturated ones, including POPC. However, the negative spontaneous curvature of diphytanoyl lipids does not allow their use in leakage techniques.

**Figure 4:**
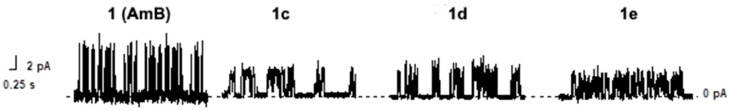
Current fluctuations corresponding to openings and closures of single channels induced by AmB, **1d**, **1c** and **1e** in the lipid bilayers composed of DPhPC/ERG (67/33 mol %) and bathed in 2.0 M KCl (pH 7.4). The transmembrane voltage was equal to 200 mV.

Figure 4 presents the electrical current fluctuations corresponding to the opening and closure of single channels formed by AmB and its tested conjugates, **1c**, **1d** and **1e**, in the lipid bilayers composed of DPhPC and ERG (67/33 mol %) and bathed in 2 M KCl (pH 7.4) at transmembrane voltage 200 mV. One can see that all tested derivatives (**1c**, **1d** and **1e**) as well as **1a [35]** produced pores of smaller amplitude than those of AmB under the same conditions. The single channel amplitude decreased in the series of AmB > **1d** > **1c** > **1e**.

**Figure 5:**
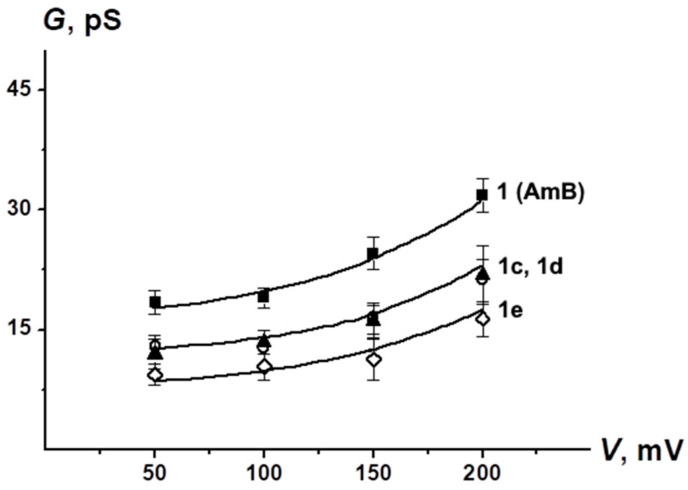
G–V curves of single channels produced by AmB, **1c**, **1d** and **1e**. Membranes were made from DPhPC/ERG (67/33 mol %) and bathed in 2.0 M KCl (pH 7.4).

Figure 5 shows G(V) diagrams of the pores produced by AmB derivatives. The chemical modification of the natural polyene molecule did not practically affect the shape of the nonlinear G(V)-dependence of the channels formed by the parent antibiotic (Figure 5). Table 4 shows the mean dwell times, τ, and the probability of polyene channels opening, P_op_.

The lifetime decreased in the order of AmB ≥ **1e** > **1d** > **1c** > **1a**. Moreover, the *P_op_* of pores induced by **1c**, **1d** and **1e** is two to three times lower than P_op_ of AmB channels. These data are in a good agreement with previously published results [35,49].

At neutral pH, single Nys channels are characterized by a low conductance that does not exceed the level of current noise of about 0.5 pA [50,51]; therefore, the registration of the single step-like transmembrane current fluctuations that might be related to single channels produced by Nys or their derivatives could not be performed at neutral pH.

**Figure 6:**
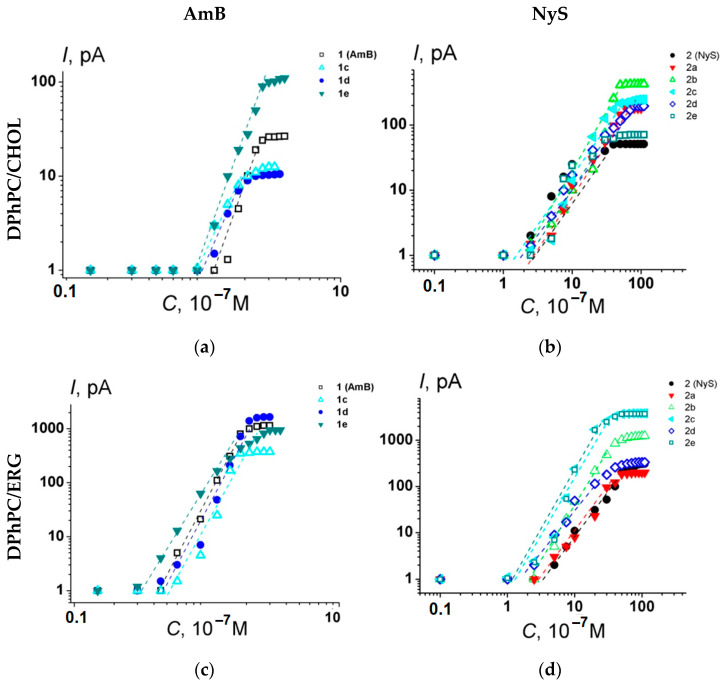
Bilogarithmic dependence of the steady-state polyene-induced transmembrane current on the concentration of AmB (**a**,**c**) and Nys (**b,d**) derivatives in the bilayer bathing solution. The transmembrane voltage was equal to 100 mV. Membranes were composed of DPhPC/CHOL (67/33 mol %) (upper panel) and DPhPC/ERG (67/33 mol %) (lower panel) and bathed in 2.0 M KCl (pH 7.4). The relation between the color symbol and the compound is given on the figure.

The one-sided addition of AmB or Nys to the membrane-bathing solution produced an increase in macroscopic conductance in a dose-dependent manner. Figure 6 shows the bilogarithmic plots of the dependences of steady-state transmembrane current flowing through membranes composed of DPhPC and CHOL or ERG at 100 mV on the concentration of the tested AmB (Figure 6a,b) and Nys derivatives (Figure 6c,d). The slopes of the linear regression of the growth section of the presented curves for AmB and NyS derivatives are close to 5 ÷ 6 and 2 ÷ 3, respectively. This means that the steady-state polyene-induced conductance is proportional to about the fourth to fifth and second to third power of the AmB and Nys derivative concentrations, respectively.

The measurements of the macroscopic ionic conductance of polyene-modified planar lipid bilayers also made it possible to estimate the *EI* value in the terms of the ratio of the threshold concentrations ($\frac{C_{\mathrm{CHOL}}}{C_{\mathrm{ERG}}}$) (Table 5). The magnitude of $\frac{C_{\mathrm{CHOL}}}{C_{\mathrm{ERG}}}$ decreased in the following series, **1d** ≥ AmB ≥ **1e** ≈ **1c** and **2c** ≈ **2e** ≥ Nys ≈ **2b** ≈ **2a** ≈ **2d**, similar to the order of EI reduction in the AmB and Nys groups presented in Table 3.

### 2.4. Comparison of Susceptibility of Ergosterol-Pathway Mutants to Polyene Derivatives

**Figure 7:**
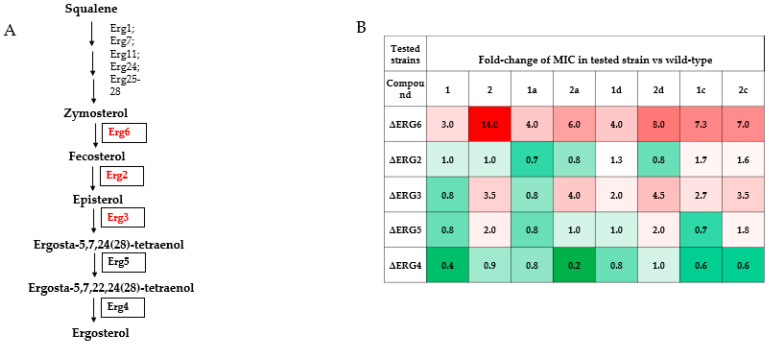
Susceptibility of ergosterol-pathway mutants to parent and derived antibiotics. Schematic of the ergosterol biosynthesis pathway (**A**) with genes implicated in clinical polyene resistance highlighted in red ([52] for Erg6, [53,54] for Erg2, [55] for Erg3) and tested genes in boxes. (**B**) Table of fold-changes in MIC of different strains, normalized to the MIC of the wild type strain of each compound. Each experiment was repeated no less than 3 times and the average result is presented.

The differences identified in the susceptibility of various fungal pathogens to the presented semisynthetic derivatives are highly relevant for their potential clinical use (Table 1); however, they are difficult to interpret in a molecular sense, because the properties of the cell wall and membrane that drive these differences are unclear. In order to gain an insight into the differences between the synthesized derivatives in a more controlled model with varying cell properties, we used mutants of the yeast *Saccharomyces cerevisiae*, which harbor specific gene-deletions in the ergosterol biosynthesis pathway. Because these cells lack ergosterol, but possess different precursors of this molecule, the fungal cells are still viable; however, their sensitivity to the polyenes changes. Differences in these mutant susceptibility patterns allow us to hypothesize that the different derivatives have a distinct affinity toward different ergosterol precursors. Notably, some of the tested gene deletions are known to cause polyene resistance in clinical fungal isolates (see legend to Figure 7), which suggests that understanding how potential novel antifungals can interact with such mutants might be relevant for planning treatment strategies.

Testing of the MICs of the various mutants showed the following patterns. Compared to wild type yeast, deletion of ERG6 showed the highest increase in relative resistance to **2**, as well as to all of the tested derivatives of **1** and **2,** with **1** showing the lowest increase in resistance. Amides **1a** and **1d** retained this property; however, **1c** showed increased resistance. All of the tested derivatives of **2** had somewhat lowered relative resistance. Note, this property might be clinically relevant, because ERG6 mutations have been implicated in clinical polyene resistance [52]. Deletion of ERG3 causes resistance to **2** and its derivatives and this resistance does not change depending on the introduced modifications. However, for **1** and its derivatives, the situation is more complex. ERG3 deletion has little effect on the resistance to **1** or **1a**, but causes some resistance to **1d** and even more so to **1c**.

Finally, the strongest increases in polyene sensitivity were observed for ERG4 deletion in response to **1**, as well as to **2a**, but were weak for the other tested polyenes.

## 3. Discussion

The development of semisynthetic derivatives of amphotericin B with an improved pharmacological profile has been underway for several decades. Among all the tested modifications, the following changes were distinguished by a significant improvement in the pharmacological properties of the polyenes: (1) the modification of the carboxylic group by amidation, esterification, reduction [17,18]; (2) the introduction of additional positive charge [17,18]; (3) the removal of the C2’-OH [56] and C35-OH groups [57] (for AmB); (4) the introduction of bulk substituents into the mycosamine group [58] and (5) the synthesis of urea [59]. The first two observations were made before the 2010s on the basis of experimental data when comparing the antifungal and hemolytic activity of a polyene antibiotic (in most cases, amphotericin) and its derivatives. The first attempt to explain the increase in the selectivity of the semisynthetic polyene derivatives was implemented using molecular dynamics methods, simulating the interaction of antibiotics with ERG- and CHOL-containing membranes. Two bis-modified AmB derivatives (esterification of carboxyl group and N-alkylation) penetrated more deeply into the bilayer with an alteration of their wobble dynamics, which in turn led to facilitated self-association in the ordered Erg-containing membranes; the ability of additional basic groups to form hydrogen bonds with phospholipids was more pronounced in ERG-containing bilayers [60]. It should be pointed out that this model might not accurately reproduce a real membrane since it was performed using dimyristoylphosphatidylcholine (DMPC) with shorter and fully saturated alkyl chains than those of phosphatidylcholines naturally occurring in biological membranes. Later, it was assumed that the decrease in toxicity in the case of AmB amides was the result of decreasing of aggregation properties [34,35]. The decrease in toxicity observed when the OH group was removed from the mycosamine was explained by the M. Burke group as a change in the native conformation of the AmB molecule, in which it is able to bind only to ergosterol. The removal of the 35-OH group from the macrocycle led to a loss of channel-forming activity, while the affinity for sterols, as well as antifungal activity, remained, so the authors proposed the “sterol-sponge model” according to which the most promising approach for obtaining non-toxic polyenes would involve diminishing pore formation while maintaining a high affinity to ergosterol. Finally, a possible explanation for the decrease in the toxicity of polyenes by modifications (4) and (5) is the destruction of the "salt bridge" between the amino group of mycosamine and the side carboxyl group of the macrocycle, stabilizing the conformation of the amphotericin molecule, providing an affinity to both cholesterol and ergosterol. It should be noted that the described studies of the causes of reducing the toxicity of semisynthetic derivatives were mainly carried out for AmB, while in the studies on the modification of nystatin, the authors of [38,58] did not perform such analyses.

The preparation of a new series of nystatin amides **2a**-**2e** and AmB amides **1c**-**1e** and their investigation in comparison to the previously described **1a** and **1b** allowed us to compare the structure–property relationships within the two major polyenes cores, Nys and AmB. Studies have shown that despite the similarity of the structures of amphotericin and nystatin, similar modifications lead to different effects. Although all modifications led to decreased zwitterionic character, the insertion of an additional positive charge and disruption of the “salt-bridge” between the C16 carboxylic group and the amino group of mycosamine, which would be expected to decrease toxicity according to known data, the observed effect on the safety of the compounds depended on both the polyene core and the diamine nature. Thus, the insertion of a short ethylenediamine moiety into the AmB and Nys cores led to more active and safe derivatives **1a** [35] and **2a**, respectively (for the last one this was shown in in vitro experiments only). However, unlike the situation with **1a**, in the case of nystatin, this effect could not be explained by the increased selectivity to ERG-containing membranes vs. CHOL-containing ones. We are more prone to attribute the result to the alteration of polyene–sterol interactions by membrane phospholipids and sphingolipids [61], although decreased aggregation properties due to the loss of amphiphilic structure might be considered as well. Insertion of the short aminoethanol group into the nystatin structure also decreased the toxicity of Nys [38]. Modification with longer diamines such as *N*,*N*-dimethylethylenediamine and *N*-methyl-1,3-propanediamine have differential effects on the two types of polyene core—the corresponding Nys amides **2c**, **2e** with antifungal activity similar to the parent antibiotic, were not toxic to human embryo kidney cells HEK293 and showed low hemolytic activity, which was well-correlated with increasing selectivity towards ERG-containing vs. CHOL-containing membranes. In contrast, AmB amides bearing the same moieties **1c** and **1e** proved to be more toxic to kidney and red blood cells, which coincided with a lower ratio of selectivity towards ERG-containing membranes. Long amide chains, both lipophilic (2-((2-fluorobenzyl)amino)ethyl)amine and hydrophilic 2-((2-aminoethyl)amino)ethanol (compounds **1b**,**d** and **2b**,**d**), led to the most pronounced increase in the permeability of CHOL- and ERG-membranes without changing the selectivity for both tested polyene cores, which might be the result of currently unidentified interactions with the lipid environment. Investigations of the single ion-permeable pores formed by AmB and its amides in ERG-containing bilayers indicated that a loss of the zwitterionic properties of the AmB molecule leads to decrease in the lifetime of the transmembrane pores and their probability to be open due to electrostatic repulsion between positively charged derivative molecules destabilizing the channel. Most likely, the introduction of a radical also affects the polarization of bonds between oxygen and hydrogen in hydroxyls in the polar chain of the lactone ring, which determines the conductance of single channels, as was shown in the case of other AmB derivatives [50]. Thus, the design of new structures should take into account not only the affinity for sterols, but also changes in the interaction of polyenes with the lipid environment, as well as the physicochemical and physicobiological properties of polyenes that affect their self-association in membranes. However, electrophysiological experiments showed that the number of polyene–sterol complexes forming the channel does not depend on the type of chemical modification of the parent molecules.

Cases of polyene resistance are recorded quite rarely. Using the example of a clinical isolate of *C. albicans* ATCC 200955, it was shown that resistance to the action of polyenes was associated with a mutation at the ERG2 locus, which caused induction in cells of a diverse array of stresses. To survive, the mutant strain requires a high level of expression of the heat shock protein Hsp90, which makes it highly sensitive to oxidative stress and temperature rise, thus making it an easier target for the immune system of an infected organism. Moreover, AmB-resistant strains have defects in the filamentation and invasion of tissues and organs. Such fungi turn out to be nonvirulent in tests on mice in vivo. [62] The main mechanism of resistance to polyenes of *Candida auris* strains, known for their resistance to all classes of antifungal drugs, is considered to be a change in ergosterol biosynthesis (mutations in the ERG2, ERG3, and ERG6 genes, overexpression of the ERG1, ERG2, ERG3, EGR5, ERG6, and ERG13 genes) [63]. To the best of our knowledge, no one has previously described the effect of chemical modifications of polyenes on the activity against mutant fungal strains that have alterations in the pathway of ergosterol biosynthesis. In this paper, it was shown that changes to the C16-carboxylic group can have considerable effects on the putative substrate specificity of polyenes toward ergosterol-pathway intermediates that are: (1) specific to the parent antifungal and (2) important for the membrane homeostasis of polyene-resistant fungal isolates. It is also possible that the observed effects are caused by general changes in the membrane properties and a complete molecular-level understanding requires further study.

Among the AmB amides obtained by our group, **1a** remains the most promising candidate for future drug-development. From the Nys series, two water-soluble amides—**2c** and **2e**—showed excellent results in tests for cell toxicity, while in the antifungal testing, **2c** was somewhat more active than **2e**. The most active Nys derivative **2a** also proved to be less toxic than the parent antibiotic, but the reason for the decreased toxicity was not clear. Nevertheless, we consider all three amides as candidates for further in vivo study.

## 4. Materials and Methods

### 4.1. General

All chemicals were purchased from commercial suppliers and used as received. Amphotericin (AmB), Nystatin (Nys), benzotriazol-1-yl-oxytripyrrolidinophosphonium hexafluorophosphate (PyBOP), dimethylsulfoxide (DMSO), calcein, KCl, 4-(2-hydroxyethyl)-1-piperazineethanesulfonic acid (HEPES), KOH, triton X-100, ethylenediamine, N,N-dimethylethylenediamine were purchased from Sigma-Aldrich (Merck KGaA, Darmstadt, Germany). N-(2-hydroxyethyl)ethylenediamine, N-(2-fluorobenzyl)ethane-1,2-diamine, N-methyl-1,3-propanediamine were purchased from abcr GmbH (Karlsruhe, Germany). Synthetic 1,2-diphytanoyl-*sn*-glycero-3-phosphocholine (DPhPC), 1-palmitoyl-2-oleoyl-*sn*-glycero-3-phosphocholine (POPC), ergosterol (ERG), and cholesterol (CHOL) were obtained from Avanti Polar Lipids (Alabaster, AL, USA). Water was distilled twice and deionized. Solutions of 2.0 or 0.1 M KCl were buffered using 5 mM HEPES-KOH at pH 7.4. Thin-layer chromatography (TLC) analysis was performed on the 0.20 mm silica gel 60 F_254_ plates (aluminum sheets 20 × 20 cm) Macherey-Nagel (Duren, Germany) in n-PrOH–EtOAc–NH_4_OH, 3:3:2. All final compounds were purified to 90%+ by reverse phase flash chromatography on C18 silica gel cartridges (Biotage SNAP) or by normal phase flash chromatography on silica gel cartridges (SNAP Ultra). The purity was assessed by reverse phase high performance liquid chromatography (HPLC) which was performed on a Shimadzu HPLC instrument of the LC 20AD series (Japan) on a Kromasil-100 C18 column (4.6 × 250 mm, particle size 5 μm, Ekzo Nobel, Sweden) with an injection volume of 20 μL (concentration of substances 0.025–0.05 mg/mL) at a flow rate of 1.0 mL/min and monitored by a diode array ultraviolet detector at 408 nm for amphotericin B derivatives and at 320 nm for nystatin derivatives. The system consisted of buffer—0.2% HCOONH_4_ at pH 4.5, 7.8, 8.4—and organic phase acetonitrile. The proportion of acetonitrile was varied from 30 to 70% for 30 min (pH 4.5, system A), 40 to 60% for 15 min (pH 8.4, system B), 20 to 60% for 30 min (pH 4.5, system C) and 35 to 60% for 15 min (pH 7.8, system D). NMR spectra were recorded on a Bruker Avance III 500 MHz NMR spectrometer with 500.23 and 125.78 MHz resonance frequencies for ^1^H and ^13^C, respectively. Spectra were recorded in DMSO-d6 solutions at 303 K and were referenced against residual solvent signals: 2.50 ppm for DMSO-d5 for ^1^H and 39.50 ppm for DMSO-d6 for ^13^C, respectively. Mixing times in the range of 10–180 ms were used to mimic stepwise magnetization propagation. For 2D TOCSY, a mixing time of 80 ms was used. For 2D ROESY and NOESY experiments, mixing times of 300 and 500 ms were used, respectively. ESI MS spectra were recorded on a Bruker microTOF-Q II instrument (BrukerDaltonics GmbH, Bremen, Germany).

### 4.2. Carboxamides of Amphotericin B and Nystatin (***1c***,***d***,***e***, ***2a***–***e***) (General Method)

AmB or Nys (200 mg, 0.22 mmol) were dissolved in DMSO (5 mL) under argon flow, and then PyBOP (172 mg, 0.33 mmol) and the corresponding diamine (1.1 mmol) were added. The reaction mixture was stirred for 1.5 h, and then Et_2_O (15 mL) was added. The mixture was stirred vigorously, then the Et_2_O layer was removed and the procedure was repeated several times until viscous oil was formed. Viscous oil was dissolved in methanol (15 mL) and then Et_2_O (100 mL) was added; the formed precipitate was filtered off, washed with Et_2_O and dried under vacuum. The progress of the reactions, chromatography purification and the purity of final compounds were monitored by TLC and HPLC analysis. The crude amide was purified by reverse-phase or normal-phase flash chromatography on Biotage SNAP C18 (10 g) and SNAP Ultra (10 g) cartridges, respectively. Reverse-phase chromatography was performed as follows: the amide was dissolved in 0.5% aq. AcOH (2 mL) or in the MeCN/0.5% aq. AcOH mixture (1:1) (2 mL) and put on a column pre-equilibrated with 0.5% aq. NH_4_OH. The elution was carried out with (A) 0.5% aq. NH_4_OH then H_2_O then 0.5% aq. AcOH and (B) acetonitrile (0→50% B). Normal-phase chromatography was performed as follows: the amide was dissolved in CH_2_Cl_2_/MeOH mixture (1:1) and put on a column pre-equilibrated with CH_2_Cl_2_:MeOH:H_2_O:CH_3_COOH mixture (64:6:1:0.1). The elution was carried out with (A) CH_2_Cl_2_ and (B) MeOH:H_2_O:CH_3_COOH (6:1:0.1) (10→35% B). Fractions containing the target compound were combined and evaporated to a small volume. The addition of Et_2_O to the solution gave the precipitate of an acetate of a targeted compound that was filtered off and dried.

Diacetate of N-(2-(N’,N’-dimethyl)aminoethyl)amide of AmB (**1c**): purified by reverse-phase chromatography; yield: 89 mg (37%); yellow powder; mp. 160–163 °C (decomp.); Rf 0.55; Rt (system A) 10.49 min; purity 93.5%; HRMS (ESI) *m*/*z*: [M + H]^+^ Calc. for C_51_H_84_N_3_O_16_^+^ 994.5846. Found 994.5904.

Diacetate of N-((2-(2-fluorophenyl-1-methyl)amino)ethyl)amide of AmB (**1d**): purified by normal-phase chromatography; yield: 60 mg (23.5%); orange powder; mp. 168–170 °C (decomp.); Rf 0.56; Rt (system A) 12.21 min; purity 91.0%; HRMS (ESI) *m*/*z*: [M + H]^+^ Calc. for C_56_H_85_FN_3_O_16_^+^ 1074.5908. Found 1074.5863.

Diacetate of N-(3-(N’-methyl)aminopropyl)amide of AmB (**1e**): purified by reverse-phase chromatography; yield: 145 mg (60%); yellow powder; mp. 155–158 °C (decomp.); Rf 0.45; Rt (system A) 10.72 min; purity 90%; HRMS (ESI) *m*/*z*: [M + H]^+^ Calc. for C_51_H_84_N_3_O_16_^+^ 994.5846. Found 994.5882.

Diacetate of N-(2-aminoethyl)amide of Nys (**2a**): purified by reverse-phase chromatography; yield: 78 mg (33%); beige powder; mp. 146–149 °C (decomp.); Rf 0.47; Rt (system B) 4.82 min; purity 96%; HRMS (ESI) *m*/*z*: [M + H]^+^ Calc. for C_49_H_82_N_3_O_16_^+^ 968,5690. Found 968,5723.

Diacetate of N-(2-((2-Hydroxyethyl)amino)ethyl)amide of Nys (**2b**): purified by reverse-phase chromatography; yield: 48 mg (22%); beige powder; mp. 138–140 °C (decomp.); Rf 0.50; Rt (system D) 8.44 min, purity 95%; HRMS (ESI) *m*/*z*: [M + H]^+^ Calc. for C_51_H_86_N_3_O_17_^+^ 1012.5952. Found 1012.6009.

Diacetate of N-(2-(N’,N’-dimethyl)aminoethyl)amide of Nys (**2c**): purified by reverse-phase chromatography; yield: 109 mg (45%); beige powder; mp. 140–143 °C (decomp.); Rf 0.53; Rt (system C) 17.76 min, purity 92%; HRMS (ESI) *m*/*z*: [M + H]^+^ Calc. for C_51_H_86_N_3_O_16_^+^ 996.6003. Found 996.5966.

Diacetate of N-((2-(2-fluorophenyl-1-methyl)amino)ethyl)amide of Nys (**2d**): purified by normal-phase chromatography; yield: 151 mg (70%); beige powder; mp. 138–140 °C (decomp.); Rf 0.53; Rt (system C) 18.68 min; purity 90%; HRMS (ESI) *m*/*z*: [M + H]^+^ Calc. for C_56_H_87_FN_3_O_16_^+^ 1076.6065. Found 1076.6062.

Diacetate of N-(3-(N’-methyl)aminopropyl)amide of Nys (**2e**): purified by reverse-phase chromatography; yield: 86 mg (36%); beige powder; mp. 134–137 °C (decomp.); Rf 0.41; Rt (system A) 10.90 min; purity 95%; HRMS (ESI) *m*/*z*: [M + H]^+^ Calc. for C_51_H_86_N_3_O_16_^+^ 996.6003. Found 996.6058.

### 4.3. Solubility Testing

For UV spectrometry, a stock solution of 4 mg/mL was prepared in pure methanol after which this solution was diluted 20-fold with water to obtain 0.2 mg/mL solutions in 5% methanol. These solutions and their sequential dilutions were used to build a calibration curve, which was then used to determine the concentration of compound in a saturated solution. These were prepared by incubating 10 mg of each sample of compound with 100 µL of deionized water for 10 min with shaking, after which the suspension was centrifuged for 1 min at 16,000× *g*, and then the supernatant was collected and centrifuged at the same speed for 20 min. The resulting supernatant was used for determining the UV absorbance. For the solutions of compounds **1a,c**,**e** and **2a**,**c**,**e**, no sediments were detected, and the concentration of the corresponding supernatants, determined by measuring the absorption of a sequentially diluted solutions, was 100 g/L. Addition of methanol to 5% did not affect the results. Calibration curve data are presented in the Supplemental Materials (Figures S16–S23).

### 4.4. Antifungal Susceptibility Testing

#### 4.4.1. Organisms

Strains of *Candida* spp. (*C. parapsilosis* 58L, *C. albicans* 604M, *C. albicans* 8R, *C. glabrata* 61L, *C. tropicalis* 3010, *C. krusei* 432M) and *Aspergillus niger* 37a, *Trichophyton rubrum* 2002 analyzed in this study were obtained from the Medical Microbiology Laboratory of the State Research Center for Antibiotics (Moscow, Russia). Cells of *Candida* spp. and spores of filamentous fungi were stored in medium supplemented with 10% (*v*/*v*) glycerol at −80 °C.

The reference strain *C. parapsilopsis* ATCC 22019 and AmB was used as control in each experiment.

The *Saccharomyces cerevisiae* strain BY4742 (*MATα his3*Δ*1 leu2*Δ*0 lys2*Δ*0 ura3*Δ*0*) and its derivatives from yeast deletion collection (EUROSCARF) were used for determining the susceptibility of ergosterol pathway mutants to the chemical samples.

#### 4.4.2. Preparation of Chemical Samples (for Pathogen Exposure)

Substances of antifungal antibiotics were dissolved in DMSO (Merck, Germany), stock solutions were prepared taking into account the activity of the antifungal pharmaceutical substance specified by the developer. The calculation of the mass of the substance or the volume of the solvent required for the preparation of the stock solution was calculated using the formulas recommended by EUCAST (European Committee on Antimicrobial Susceptibility Testing). Dilutions were prepared according to ISO 20776-1:2006. Working solutions were prepared with a final concentration of 0.125–64 mg/mL. All of the test compounds were dissolved to concentrations of 10 mg/mL in 100% DMSO (Merck, Darmstad, Germany). The dissolved stock concentration chemical compounds were then diluted in the growth medium RPMI1640 (Merck) to obtain an initial concentration of 64 μg/mL (L+), which was then directly serially diluted before testing of MIC. The final range concentrations of the test compounds was 0.015–32 μg/mL.

The 24 h-old cultures of *Candida* spp. and ~3 week culture of *Trichophyton* and *Aspergilli* grown in Sabourau dextrose agar (Sigma-Aldrich, St. Louis, MO, USA) were used to evaluate sensitivity to antimicrobial agents. For inoculum preparation of filamentous fungi, part of the colony was transferred to sterile distilled water, containing 0.1% (*v*/*v*) Tween 80 and shaken vigorously. The suspension was filtered through sterile gauze and the number of spores/conidia was counted using a hemocytometer, as a stock spore solution for inoculum. Antifungal susceptibility testing was performed by EUCAST broth microdilution according to the E.DEF 7.3.2 and E.DEF 9.3.2 methods in the liquid nutrient medium PRMI 1640 with 2% (*m*/*v*) glucose, with L-glutamine and without bicarbonate (Merck, Germany). MOPS (Sigma-Aldrich, USA) was used as a buffer to this medium at a final concentration of 0.165 mol/L at pH 7.0. Minimum inhibitory concentration (MIC) was defined as the lowest concentration that resulted in complete growth inhibition after incubation for 24, 48 h for *Candida* spp., 48–72 h for dermatophytes.

For routine internal quality control when assessing sensitivity to antifungal drugs (MIC), a control test strain of *Candida parapsilosis* ATCC 22019, recommended by EUCAST, was used. Target values of the MIC for amphotericin: 0.25–0.5; allowable values: 0.125–1.0 mg/mL. To determine the sensitivity of pathogenic yeasts, an 18–48 h yeast culture grown at a temperature of 35 ± 2 °C under aerobic conditions on non-selective nutrient agar (Sabouraud agar) was used. The final concentration of the inoculum was 0.5–2.5 × 10^5^ CFU/mL. Microdilution plates were incubated at 35 ± 2 °C under aerobic conditions for 24 ± 2 h.

The study was carried out by the method of serial microdilutions in a liquid nutrient medium. For the fungal pathogens, to set up the experiment, we used liquid nutrient medium RPMI 1640 with 2% (*m*/*v*) glucose, with L-glutamine and without bicarbonate (Merck, Germany). MOPS (Sigma-Aldrich, USA) was used as a buffer to this medium at a final concentration of 0.165 mol/L at pH 7.0. The studies were performed in sterile disposable 96-well plates with flat-bottomed wells with a capacity of 300 µL (Corning, Somerville, MA, USA).

For *S. cerevisiae*, cells from a 12 h culture in YPD (1% yeast extract (*w*/*v*), 2% peptone (*w*/*v*), 2% glucose (*w*/*v*)) medium were inoculated into fresh YPD supplemented with 2-fold differing concentrations of tested substances in DMSO, and a constant concentration (0.05% *v*/*v*) of DMSO (Sigma) in 96-well plates (200 µL final volume), at an initial OD600 of 0.05. Cell growth was judged after 24 h of growth at 30 °C with shaking, with the MIC being the lowest concentration of drug where no growth was observed.

### 4.5. Cell Culture and Antiproliferative Activity

HEK293 cells (from ATCC) were propagated in Dulbecco’s modified Eagle’s medium supplemented with 5% fetal calf serum, 2 mM L-glutamine, 100 units/mL penicillin and 100 μg/mL streptomycin at 37 °C, 5% CO_2_ in a humidified atmosphere. Cells in logarithmic phase of growth were used in the experiments. Tested compounds were dissolved in DMSO as 10 mM stock solutions followed by serial dilutions with medium immediately before experiments. The cytotoxicity was determined in a formazan conversion assay (3-(4,5-dimethylthiazol-2-yl)-2,5-diphenyltetrazolium bromide (MTT) test. Briefly, cells (5 × 10^3^ in 190 μL of culture medium) were plated into a 96-well plate (Becton Dickinson, Franklin Lakes, NJ, USA) and treated with 0.1% DMSO (vehicle control) or with the tested compounds (0.10–1000 μM; each concentration in duplicate) for 72 h. After completion of drug exposure, 50 μg of MTT was added to each well for an additional 2 h. Formazan was dissolved in DMSO, and the absorbance at 540 nm was measured. Cell viability at a given drug concentration (% MTT conversion) was calculated as the percentage of absorbance in wells with drug-treated cells to absorbance in wells with DMSO-treated cells (100%).

### 4.6. Testing of Hemolysis Activity

Briefly, erythrocytes were isolated from human blood. The blood was incubated at 4 °C (2–3 h). Erythrocyte suspension (100 µL) was diluted in physiological buffer (pH 7.2) to a total volume of 500 µL. Solutions of the compounds under investigation with an initial concentration of 10 mM (DMSO) were diluted with phosphate-buffered saline (PBS) buffer (1:10). The resulting solutions were added to Eppendorf tubes in the amount necessary to create the test concentration (10, 20 and 50 µM) and a mixture of PBS with erythrocytes was added to a total volume of 200 µL. The mixture of red blood cells with 1% triton X-100 was used as a positive control. Intact control solution was a mixture of erythrocytes with PBS and solvent (DMSO). Probes were incubated for 1 h (additionally for AmB (**1**), Nys (**2**), **2c** and **1d** maximum 24 h) at 37 °C, and centrifuged at 252 relative centrifugal force (RCF) for 1.5 min, and the supernatants were transferred to a 96-well plate. The optical density of supernatants was measured on a microplate spectrophotometer (BioTek, ELx800, Winooski, VT, USA) at 570 nm. The percentages of hemolysis were calculated relative to positive control, which was taken as 100%.

### 4.7. Calcein Release from Large Unilamellar Vesicles

The fluorescence of calcein released from large unilamellar vesicles was used to monitor the membrane permeabilization induced by different polyenes. Liposomes were prepared from POPC/CHOL (67/33 mol %) and POPC/ERG (67/33 mol %) by extrusion using Avanti Polar Lipids (Alabaster, AL, USA) as described in previous work [49]. Polyenes (AmB (**1**), Nys (**2**) and their derivatives) from stock solution (10 mM in DMSO or H_2_O) were added to calcein-loaded liposomes. Time-dependent increase in fluorescence of released calcein induced by 5 μM of antibiotics was measured during 30–60 min.

The degree of calcein release was determined at 25 °C using a “Fluorat-02-Panorama” spectrofluorometer (Lumex, Saint-Petersburg, Russia). The excitation wavelength was 490 nm and the emission wavelength was 520 nm. The detergent triton X-100 (at a final concentration of 1%) was added at the end of each experiment for complete disruption of liposomes (leading to complete release of calcein and maximal fluorescence).

The relative intensity of calcein fluorescence (RF, %) was used to describe the dependence of the permeabilization of the liposomes by various polyenes. RF was calculated using the following Formula (1):

(1) $$\mathrm{RF}=\frac{I\;-{\;I}_{0}}{I_{\max}/0.9-{\;I}_{0}}\cdot 100\%$$

where I and I_0_ were the calcein fluorescence intensities in the sample in the presence and in the absence of AmB, Nys and their derivatives, respectively, and I_max_ was the maximal fluorescence of the sample after lysis of liposomes by triton X-100. The factor of 0.9 was introduced to calculate the dilution of the sample by triton X-100.

Time series of calcein release were fitted with two-exponential functions with characteristic times *t*_1_ and *t*_2_ related to fast and slow components of calcein release.

The values of *RF*, *t*_1_ and *t*_2_ were presented as mean ± s.e. (*p* ≤ 0.05).

### 4.8. Registration of Ion Channels in Planar Lipid Bilayers

Virtually solvent-free planar lipid bilayers were prepared according to the monolayer-opposition technique [64] on a 50 μm diameter aperture in a 10 μm thick Teflon film separating two (*cis* and *trans*) compartments of the Teflon chamber. The aperture was pretreated with hexadecane. The lipid bilayers were made from DPhPC/ERG (67/33 mol %). After the membrane was completely formed and stabilized, AmB and its derivatives from a stock solution (1 mM in DMSO or H_2_O) were added to *cis*-compartments to obtain the different concentrations presented in Figure 5. Ag/AgCl electrodes with agarose/2 M KCl bridges were used to apply a transmembrane voltage and measure the transmembrane current. “Positive voltage" refers to the case in which the *cis*-side compartment was positive with respect to the *trans*-side. All experiments were performed at room temperature. The final concentration of solvent in the chamber did not exceed 10^−4^ mg/mL and did not produce any changes in the stability and conductance of the bilayers.

Current measurements were performed using an Axopatch 200B amplifier (Molecular Devices, San Jose, CA, USA) in voltage clamp mode. Data were digitized by a Digidata 1440A and analyzed using a pClamp 10 (Molecular Devices, San Jose, CA, USA) and Origin 7.0 (OriginLab Corporation, Northampton, MA, USA). Data were acquired at a sampling frequency of 5 kHz using low-pass filtering at 1 kHz, and the current tracks were processed through an 8-pole Bessel 100-kHz filter.

Single-channel conductance (G) was defined as the ratio between the current flowing through a single polyene channel (i) and transmembrane potential (V). The total numbers of events used for the channel conductance fluctuation and dwell time (τ) analysis were 500 ÷ 1000 and 1500 ÷ 2000, respectively. The probability of the polyene channel to be in an open state (P_op_) was calculated using the following Formula (2):

(2) $$P_{\mathrm{op}}\;=\;\;\frac{\tau }{\tau \;+\;\tau_{\mathrm{close}}}$$

where τ is the dwell time of the single polyene channel and τ_close_ is the time that the channel is in a closed state.

The values of the threshold polyene antibiotic concentrations (C_CHOL/ERG_) were determined by the intersections of the straight line fitting of the initial and growth portion sections of the bilogarithmic plots of the dependences of steady-state transmembrane current flowing through polyene-modified membranes at 100 mV on the concentration of the tested derivatives.

The values of *G* were presented as mean ± s.d. (*p* ≤ 0.05); τ, P_op_, and **C_CHOL/ERG_** were presented as mean ± s.e. (*p* ≤ 0.05).

## Figures and Tables

**Table 1:** The antifungal activity of the semisynthetic polyene antibiotic derivatives **1a**–**1e**, **2a**–**2e** in comparison with AmB (**1**) and Nys (**2**).

Minimum Inhibitory Concentration (MIC, µg/mL)												
Tested Strains	Compound											
	1a ^1^	2a	1b ^1^	2b	1c	2c	1d	2d	1e	2e	1	2
*C. parapsilosis* ATCC 22019	0.03/0.06 ^2^	0.5/1	0.06/0.125	2/4	1/1	1/2	2/2	2/4	1/2	1/2	1/2	2/2
*C. albicans* ATCC 10231	n/t	1/2	n/t	2/4	1/1	2/4	2/2	2/4	1/1	4/8	0.5/0.5	1/2
*A. fumigatus* ATCC 46645	n/t	2	n/t	4	1	2	4	2	2	8	2	4
*A. niger* 37a	0.125	8	0.5	16	4	8	1	32	2	16	2	8
*T. rubrum* 2002	0.5	8	0.25	32	2	8	1	8	2	8	1	8
*C. krusei* 432M	0.25/0.5	4/4	0.25/0.5	8/8	2/2	16/16	2/2	8/8	2/2	8/16	0.5/1.0	4/4
*C. albicans* 604M	0.05/0.1	1/1	n/t	2/4	1/1	2/2	2/2	2/2	1/1	2/4	1/1	1/2
*C. albicans* 8R	0.05/0.1	1/2	n/t	4/4	1/1	2/4	2/2	4/4	1/1	2/4	1/1	1/2
*C. glabrata* 61L	0.03/0.06	1/1	n/t	2/2	1/1	1/2	2/4	2/2	2/2	2/2	1/2	1/2
*C. tropicalis* 3010	n/t	1/1	n/t	2/2	0.5/1	2/2	2/2	2/2	1/1	2/4	0.5/1	1/2
*C. parapsilosis* 58L	n/t	1/2	n/t	2/4	1/1	1/2	2/2	2/4	1/1	2/2	0.5/1	1/2

^1^ Published earlier in [35]; ^2^ 24/48 h.

**Table 2:** The antiproliferative activity of the semisynthetic polyene antibiotic derivatives **1a**–**1e**, **2a**–**2e** in comparison with AmB (**1**), Nys (**2**) and Doxorubicin.

Compound	IC_50_, μM (HEK293)
**1a**	35.0 ± 4.0
**1b**	4.0 ± 0.4
**1c**	3.9 ± 0.5
**1d**	20.0 ± 2.4
**1e**	6.0 ± 0.7
**2a**	47.0 ± 5.2
**2b**	47.0 ± 4.7
**2c**	>50.0
**2d**	15.0 ± 1.7
**2e**	>50.0
**1** (AmB)	18.0 ± 4.0
**2** (Nys)	15.0 ± 3.0
Doxorubicin	0.20 ± 0.02

**Table 3:** The characteristic parameters of the dependence of calcein release from large unilamellar vesicles of indicated composition induced by 5 μM of antibiotic on time.

	POPC/CHOL (67/33 mol %)			POPC/ERG (67/33 mol %)			EI ^3^
Polyene	RF_max_^1^, %	t_1_^2^, min	t_2_^2^, min	RF_max_, %	t_1_, min	t_2_, min	
AmB (**1**)	6 ± 1	0.6 ± 0.3	12.4 ± 8.1	9 ± 1	0.3 ± 0.1	5.8 ± 1.1	1.5 ± 0.4
**1c**	13 ± 1	0.6 ± 0.1	7.9 ± 0.4	16 ± 2	0.5 ± 0.2	6.9 ± 1.1	1.2 ± 0.2
**1d**	39 ± 3	0.5 ± 0.1	13.1 ± 4.8	57 ± 3	0.4 ± 0.1	11.9 ± 1.2	1.6 ± 0.2
**1e**	16 ± 1	0.3 ± 0.1	4.7 ± 2.6	21 ± 3	0.1 ± 0.1	9.7 ± 2.4	1.3 ± 0.2
Nys (**2**)	10 ± 2	0.9 ± 0.3	5.7 ± 1.9	19 ± 3	0.7 ± 0.1	5.4 ± 0.5	1.9 ± 0.8
**2a**	33 ± 5	0.5 ± 0.2	4.2 ± 0.2	44 ± 2	0.5 ± 0.1	4.8 ± 0.6	1.3 ± 0.3
**2b**	71 ± 7	0.5 ± 0.1	4.6 ± 0.3	82 ± 5	0.5 ± 0.1	4.3 ± 0.4	1.3 ± 0.3
**2c**	37 ± 9	0.2 ± 0.1	2.4 ± 0.2	86 ± 8	0.3 ± 0.1	6.6 ± 2.4	2.3 ± 0.8
**2d**	39 ± 9	0.3 ± 0.1	3.9 ± 0.3	70 ± 6	0.5 ± 0.1	7.9 ± 2.4	1.8 ± 0.6
**2e**	22 ± 4	0.9 ± 0.4	6.7 ± 0.4	75 ± 7	0.4 ± 0.1	2.9 ± 0.7	3.4 ± 0.9

^1^ RF_max_—maximal leakage of calcein from unilamellar vesicles composed of POPC/CHOL and POPC/ERG respectively; ^2^ t_1_, t_2_—characteristic times referring to fast and slow components, respectively, of two exponential dependence fitting the time dependence of calcein release ^3^ EI—the efficiency index determined as a ratio of the RF_max_ from unilamellar vesicles composed of POPC/ERG and POPC/CHOL.

**Table 4:** Characteristic parameters of the single ion-permeable pores induced by different AmB derivatives in DPhPC/Erg (67/33 mol %) bilayers.

Polyene	G_200 mV_^1^, pS	τ ^2^*,* ms	P_op_^3^
**1** (AmB) ^4^	31.7 ± 2.1	23 ± 4	0.49 ± 0.09
**1a** ^4^	20.8 ± 1.8	6 ± 2	0.23 ± 0.07
**1c**	21.3 ± 3.2	10 ± 1	0.24 ± 0.11
**1d**	21.2 ± 2.6	11 ± 1	0.14 ± 0.08
**1e**	16.4 ± 2.2	17 ± 2	0.29 ± 0.12

^1^ G_200 mV_—the mean conductance of polyene channels at 200 mV. ^2^ τ—the mean dwell time of polyene pores. ^3^ P_op_—the probability of polyene channels being open. ^4^—according to [35].

**Table 5:** The characteristic parameters of the dependences of macroscopic ionic conductance induced by different AmB and Nys derivatives in CHOL- and ERG- enriched bilayers on the concentration of polyenes.

Polyene	C_CHOL_^1^, 10^−7^ M	m_CHOL_^2^	C_ERG_^1^, 10^−7^ M	m_ERG_^2^	C_CHOL_/C_ERG_
AmB (**1**)	0.98 ± 0.17	5–6	0.67 ± 0.16	6–6	1.5 ± 0.6
**1c**	0.88 ± 0.08	5–5	0.98 ± 0.07	4–5	0.9 ± 0.1
**1d**	0.94 ± 0.11	4–5	0.51 ± 0.09	5–6	1.8 ± 0.5
**1e**	1.05 ± 0.05	4–5	0.92 ± 0.09	5–6	1.1 ± 0.2
Nys (**2**)	1.9 ± 0.2	2–3	1.8 ± 0.1	2–3	1.1 ± 0.2
**2a**	2.2 ± 0.3	2–2	2.1 ± 0.6	2–2	1.0 ± 0.4
**2b**	1.7 ± 0.1	3–3	1.6 ± 0.2	2–3	1.1 ± 0.2
**2c**	3.1 ± 0.2	2–2	2.0 ± 0.4	2–3	1.6 ± 0.4
**2d**	2.9 ± 0.5	2–3	2.7 ± 0.8	2–2	1.0 ± 0.5
**2e**	2.4 ± 0.2	2–3	1.6 ± 0.3	2–3	1.5 ± 0.4

^1^ C_CHOL_, C_ERG_—the threshold concentration of polyenes in the solution bathing lipid bilayers composed of DPhPC/CHOL (67/33 mol %) and DPhPC/ERG (67/33 mol %), respectively; ^2^ m_CHOL_, m_ERG_—the slopes of the linear regression of the growth section of lgI(lgC) dependence characterizing the number of pore forming complexes of the polyene and CHOL or ERG, respectively.

## Data Availability

Not applicable.

## Supplementary Materials

The following supporting information can be downloaded at: https://www.mdpi.com/article/10.3390/antibiotics12010151/s1: Table S1: ^1^H and ^13^C spectra assignment for derivatives **1c**–**1e**, **2a**–**2e**; Figures S1–S15: ^1^H, ^13^C NMR spectra of the derivatives **1c**–**1e**, **2a**–**2e**; Figures S16–S23: Calibration curves (solubility in water).
