# Supplementary material for: Semisynthetic Amides of Amphotericin B and Nystatin A1: A Comparative Study of In Vitro Activity/Toxicity Ratio in Relation to Selectivity to Ergosterol Membranes
Source: Antibiotics (Basel). 2023 Jan 11;12(1):151. doi: 10.3390/antibiotics12010151 (PMC9854944; doi:10.3390/antibiotics12010151)
Supplement: Supplementary file 1 [file antibiotics-12-00151-s001.zip › antibiotics-2103766-supplementary.pdf]

SUPPORTING INFORMATION

Semisynthetic amides of amphotericin B and nystatin A1: a comparative study of in vitro activity/toxicity ratio in relation to selectivity to ergosterol membranes

Anna Tevyashova [1,*], Svetlana Efimova [2], Alexander Alexandrov [3], Olga Omelchuk [1], Eslam Ghazy [3,4,5], Elena Bychkova [1], Georgy Zatonsky [1], Natalia Grammatikova [1], Lyubov Dezhenkova [1], Svetlana Solovieva [1], Olga Ostroumova [2], and Andrey Shchekotikhin [1]

[1] Gause Institute of New Antibiotics, 11 B. Pirogovskaya, Moscow, 199021, Russia
[2] Institute of Cytology of the Russian Academy of Sciences, 4 Tikhoretsky ave., St. Petersburg, 194064, Russia
[3] Federal Research Center "Fundamentals of Biotechnology" of the Russian Academy of Sciences, Bach Institute of Biochemistry, 33 Leninsky Ave., bld. 2, Moscow, 119071, Russia
[4] Institute of biochemical technology and nanotechnology, Peoples' Friendship University of Russia (RUDN), 6 Miklukho-Maklaya Street, 17198, Moscow, Russia
[5] Department of microbiology, Faculty of Pharmacy, Tanta University, Tanta 31111, Egypt
*Correspondence: chulis@mail.ru; Tel.: +7-499-246-06-36

LIST OF CONTENTS

Table S1. ^{1}H and ^{13}C spectra assignment for derivatives **1c-1e**, **2a-2e** 2

Figures S1-S15. ^{1}H, ^{13}C NMR spectra of the derivatives **1c-1e**, **2a-2e**...................... 8

Figures S16-S20. Calibration curves (solubility in water). 23

Table S1. ^{1}H and ^{13}C spectra assignment for amphotericin derivatives **1c-1e**, **2a-2e**

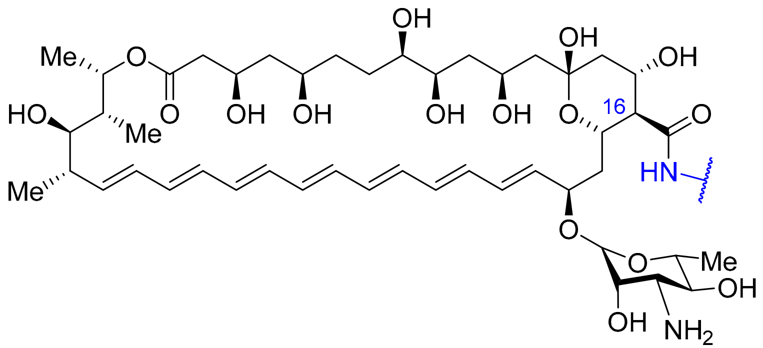

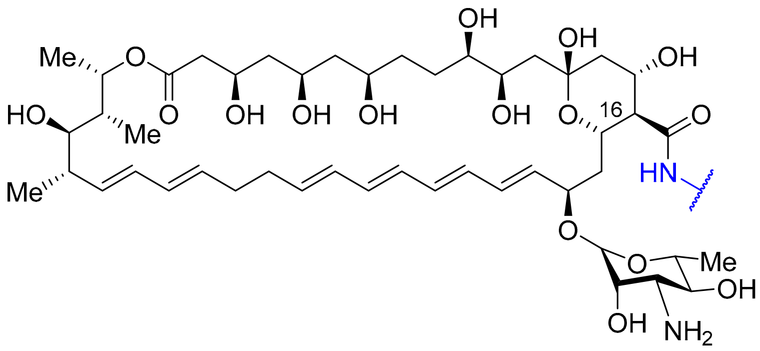

Compound. $^{13}C/^{1}H$ δ. ppm									
**Atom**	**1c**	**1d**	**1e**	**Atom**	**2a**	**2b**	**2c**	**2d**	**2e**
**Aglycone**									
**1 O-C(O)**	170.5<br>-	170.5<br>-	170.5<br>-	**1 O-C(O)**	170.4<br>-	170.4<br>-	170.3<br>-	170.4<br>-	170.3<br>-
**2 CH_2**	41.9<br>2.18	42.0<br>2.20	42.1<br>2.19	**2 CH_2**	42.5<br>2.34; 2.30	42.5<br>2.35; 2.30	42.6<br>2.34; 2.29	42.6<br>2.34; 2.28	42.6<br>2.35; 2.29
**3 CH**	66.1<br>4.06	66.1<br>4.07	66.2<br>4.08	**3 CH**	65.8<br>4.02	65.8<br>4.02	65.8<br>4.01	65.8<br>4.02	65.5<br>4.03
**4 CH_2**	44.6<br>1.40; 1.35	44.7<br>1.44; 1.37	44.7<br>1.42; 1.35	**4 CH_2**	44.2<br>1.49	44.2<br>1.49	44.4<br>1.48; 1.40	44.2<br>1.48	44.2<br>1.48
**5 CH**	69.1<br>3.53	69.1<br>3.56	69.2<br>3.55	**5 CH**	67.9<br>3.82	67.9<br>3.80	69.4<br>3.60	67.9<br>3.80	68.0<br>3.81
**6 CH_2**	34.9<br>1.42; 1.32	34.9<br>1.42; 1.32	35.0<br>1.42; 1.31	**6 CH_2**	34.3<br>1.47; 1.39	34.3<br>1.49; 1.38	34.4<br>1.47; 1.37	34.4<br>1.47; 1.37	34.4<br>1.47; 1.39
**7 CH_2**	28.9<br>1.58; 1.29	28.9<br>1.60; 1.31	29.0<br>1.58; 1.29	**7 CH**	69.4<br>3.61	69.4<br>3.59	67.9<br>3.80	69.4<br>3.59	69.3<br>3.62

**8 CH**	73.5<br>3.11	73.5<br>3.14	73.6<br>3.13	**8 CH_2**	42.2<br>1.68; 1.57	42.2<br>1.68; 1.57	44.4<br>1.47; 1.40	42.2<br>1.68; 1.57	42.2<br>1.67; 1.57
**9 CH**	73.7<br>3.49	73.7<br>3.51	73.7<br>3.48	**9 CH_2**	28.4<br>1.45	28.4<br>1.45	28.4<br>1.45	28.4<br>1.44	28.4<br>1.44
**10 CH_2**	39.5<br>1.59; 1.36	39.5<br>1.59; 1.36	39.7<br>1.58; 1.34	**10 CH**	73.3<br>3.25	73.2<br>3.28	73.3<br>3.25	73.3<br>3.25	73.3<br>3.26
**11 CH**	67.7<br>4.24	67.7<br>4.27	67.7<br>4.26	**11 CH**	70.0<br>3.92	70.1<br>3.91	70.0<br>3.91	70.0<br>3.91	70.0<br>3.91
**12 CH_2**	46.2<br>1.55	46.1<br>1.59	46.3<br>1.56	**12 CH_2**	44.4<br>1.48; 1.40	44.3<br>1.48; 1.40	44.4<br>1.48; 1.40	44.4<br>1.47; 1.40	44.4<br>1.47; 1.40
**13 C-O**	97.0<br>-	97.1<br>-	97.0<br>-	**13 C-O**	97.1<br>-	97.0<br>-	97.0<br>-	97.1<br>-	97.1<br>-
**14 CH_2**	44.4<br>1.88; 1.09	44.5<br>1.93; 1.12	44.7<br>1.89; 1.11	**14 CH_2**	44.6<br>1.91; 1.16	44.5<br>1.91; 1.15	44.5<br>1.91; 1.15	44.6<br>1.91; 1.16	44.7<br>1.90; 1.15
**15 CH**	64.8<br>4.01	64.9<br>4.04	64.7<br>4.05	**15 CH**	64.7<br>4.00	64.7<br>4.00	64.7<br>3.99	64.7<br>4.00	64.6<br>4.00
**16 CH**	57.0<br>1.89	57.0<br>1.92	56.9<br>1.90	**16 CH**	57.2<br>1.96	57.3<br>1.92	57.3<br>1.91	57.3<br>1.93	57.3<br>1.90
**17 HC-O**	65.0<br>4.23	64.9<br>4.28	65.3<br>4.23	**17 HC-O**	65.9<br>3.95	65.8<br>3.95	65.8<br>3.95	65.8<br>3.95	65.5<br>3.89
**18 CH_2**	36.2<br>1.95; 1.45	36.3<br>1.96; 1.53	36.6<br>1.97; 1.48	**18 CH_2**	37.5<br>1.68; 1.59	37.4<br>1.68; 1.60	37.4<br>1.67; 1.61	37.4<br>1.67; 1.59	37.5<br>1.67; 1.58
**19 CH-O**	74.2<br>4.36	74.6<br>4.37	74.6<br>4.36	**19 CH-O**	75.7<br>4.30	76.0<br>4.30	75.5<br>4.30	75.6<br>4.29	75.8<br>4.29
**20 CH**	136.3<br>5.97	136.2<br>6.00	136.7<br>5.97	**20 CH**	134.0<br>5.65	134.1<br>5.65	134.1<br>5.66	134.0<br>5.64	134.1<br>5.64

**21 CH**	128.5<br>6.09	128.9<br>6.14	129.0<br>6.10	**21 CH**	130.9<br>6.19	130.9<br>6.20	130.7<br>6.20	130.8<br>6.19	131.0<br>6.20
**22 CH**	133.2<br>6.31	133.0<br>6.33	133.1<br>6.33	**22 CH**	132.7<br>6.23	133.0<br>6.20	132.5<br>6.23	133.0<br>6.20	132.7<br>6.24
**23 CH**	132.1<br>6.29	132.0<br>6.29	132.0<br>6.29	**23 CH**	131.8<br>6.23	132.0<br>6.24	131.9<br>6.23	131.8<br>6.23	132.0<br>6.24
**24 CH**	133.5<br>6.39	133.6<br>6.47	133.8<br>6.46	**24 CH**	130.8<br>6.20	130.9<br>6.21	130.8<br>6.20	130.8<br>6.21	130.8<br>6.21
**25 CH**	132.3<br>6.32	132.2<br>6.34	132.2<br>6.34	**25 CH**	133.0<br>6.20	133.1<br>6.20	132.9<br>6.20	133.1<br>6.21	133.0<br>6.20
**26 CH**	133.5<br>6.39	133.2<br>6.40	133.1<br>6.32	**26 CH**	131.0<br>6.11	131.0<br>6.11	131.0<br>6.11	131.0<br>6.11	131.1<br>6.10
**27 CH**	132.4<br>6.29	132.4<br>6.32	132.3<br>6.30	**27 CH**	134.2<br>5.67	134.3<br>5.67	134.2<br>5.68	134.3<br>5.68	134.3<br>5.67
**28 CH**	133.5<br>6.35	133.5<br>6.35	133.6<br>6.35	**28 CH_2**	31.6<br>2.17	31.7<br>2.15	31.6<br>2.18	31.6<br>2.19	31.6<br>2.18
**29 CH**	132.1<br>6.31	132.2<br>6.33	132.2<br>6.33	**29 CH_2**	31.6<br>2.17	31.7<br>2.15	31.6<br>2.18	31.6<br>2.19	31.6<br>2.18
**30 CH**	131.8<br>6.16	131.6<br>6.18	131.8<br>6.17	**30 CH**	131.1<br>5.50	131.1<br>5.50	131.1<br>5.51	131.1<br>5.49	131.0<br>5.49
**31 CH**	132.2<br>6.17	132.2<br>6.19	132.1<br>6.17	**31 CH**	131.1<br>5.95	131.0<br>5.97	131.2<br>5.96	131.2<br>5.96	131.2<br>5.95
**32 CH**	128.5<br>6.09	128.9<br>6.13	128.6<br>6.11	**32 CH**	129.3<br>5.96	129.4<br>5.97	129.3<br>5.96	129.3<br>5.96	129.4<br>5.97

**33 CH**	136.7<br>5.44	136.8<br>5.48	136.7<br>5.45	**33 CH**	135.5<br>5.52	135.5<br>5.52	135.5<br>5.52	135.5<br>5.52	135.5<br>5.51
**34 CH**	42.2<br>2.29	42.3<br>2.29	42.3<br>2.29	**34 CH**	40.3<br>2.25	40.3<br>2.25	40.3<br>2.25	40.3<br>2.25	40.3<br>2.26
**35 CH_3**	18.3<br>1.03	18.4<br>1.05	18.4<br>1.05	**35 CH_3**	16.6<br>0.96	16.6<br>0.96	16.6<br>0.96	16.6<br>0.96	16.6<br>0.96
**36 CH**	77.1<br>3.11	77.1<br>3.11	77.0<br>3.11	**36 CH**	75.9<br>3.14	75.9<br>3.14	75.9<br>3.14	75.9<br>3.14	75.9<br>3.14
**37 CH**	39.6<br>1.73	39.7<br>1.75	39.7<br>1.75	**37 CH**	39.9<br>1.81	39.9<br>1.81	39.8<br>1.81	39.9<br>1.80	39.9<br>1.80
**38 CH_3**	11.9<br>0.91	12.0<br>0.93	11.9<br>0.93	**38 CH_3**	12.0<br>0.86	12.0<br>0.86	12.0<br>0.89	12.0<br>0.86	12.0<br>0.86
**39 CH**	68.8<br>5.21	68.9<br>5.22	68.9<br>5.22	**39 CH**	70.4<br>5.08	70.5<br>5.08	70.4<br>5.08	70.5<br>5.08	70.5<br>5.08
**40 CH_3**	16.8<br>1.11	16.8<br>1.11	16.9<br>1.13	**40 CH_3**	16.5<br>1.10	16.4<br>1.10	16.4<br>1.10	16.4<br>1.10	16.4<br>1.10
**41 C=O**	172.6<br>-	172.5<br>-	171.9<br>-	**41 C=O**	172.0<br>-	171.8<br>-	171.7<br>-	171.8<br>-	171.9<br>-
**Mycosamine**									
**1' CH**	96.8<br>4.31	95.5<br>4.38	97.1<br>4.30	**1' CH**	98.4<br>4.36	98.5<br>4.36	98.5<br>4.35	98.3<br>4.36	98.8<br>4.34
**2' CH**	69.6<br>3.61	67.0<br>3.88	69.6<br>3.62	**2' CH**	69.4<br>3.61	69.8<br>3.60	70.1<br>3.57	69.5<br>3.64	70.0<br>3.58
**3' CH**	56.6<br>2.39	55.4<br>2.88	56.5<br>2.40	**3' CH**	56.3<br>2.47	56.4<br>2.43	58.2<br>2.28	56.3<br>2.50	56.6<br>2.37

**4' CH**	68.5<br>3.28	68.5<br>3.28	72.8<br>2.93	**4' CH**	72.3<br>2.95	72.6<br>2.92	73.1<br>2.88	72.3<br>2.96	73.0<br>2.89
**5' CH**	72.9<br>2.92	72.9<br>3.12	73.2<br>3.04	**5' CH**	73.0<br>3.04	73.0<br>3.04	73.0<br>3.02	73.0<br>3.03	73.1<br>3.02
**6' CH_3**	17.8<br>1.14	17.5<br>1.18	18.0<br>1.13	**6' CH_3**	17.9<br>1.12	17.9<br>1.12	18.0<br>1.12	17.9<br>1.11	18.0<br>1.11
**Amide moiety**									
**NH**	7.84	8.09	8.06	**NH**	8.06	7.86	7.78	7.87	7.93
	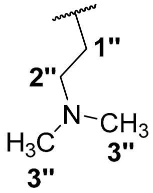	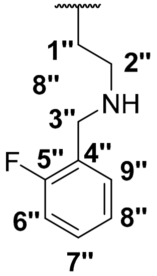	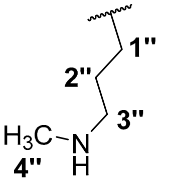		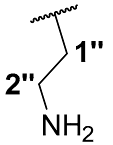	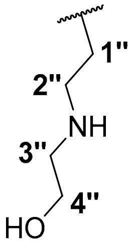	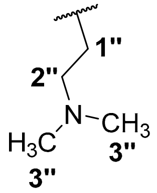	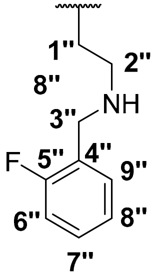	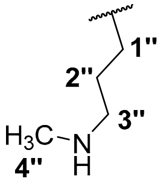
**1′′**	36.8<br>*3.19; 3.11*	68.5<br>3.29	36.4<br>3.16; 3.08	**1′′**	40.5<br>3.14	38.4<br>3.15	36.7<br>3.16	38.4<br>3.18	36.5<br>3.09
**2′′**	58.5<br>*2.29*	55.4<br>*2.89*	28.1<br>1.62	**2′′**	40.5<br>2.63	48.5<br>2.59	58.2<br>2.27	48.1<br>2.57	28.6<br>1.56
**3′′**	45.5<br>*2.14*	44.3<br>*4.04*	48.0<br>2.59	**3′′**		51.2<br>2.59	45.1<br>2.14	45.4<br>3.73	48.5<br>2.53
**4′′**		124.8<br>-	34.9<br>2.32	**4′′**		60.2<br>3.44		127.4<br>(J_{CF}=15.3 Hz)<br>-	35.5<br>2.29

**5′′**		161.4; 159.5<br>(J_{CF}=146.5<br>Hz)<br>-						160.3<br>(J_{CF}=243.6<br>Hz<br>-	
**6′′**		115.3<br>7.26						114.8<br>(J_{CF}=21.8 Hz)<br>7.13	
**7′′**		131.4<br>7.54						128.4<br>7.28	
**8′′**		124.7<br>7.27						124.1<br>7.17	
**9′′**		130.5<br>7.43						130.2<br>7.46	

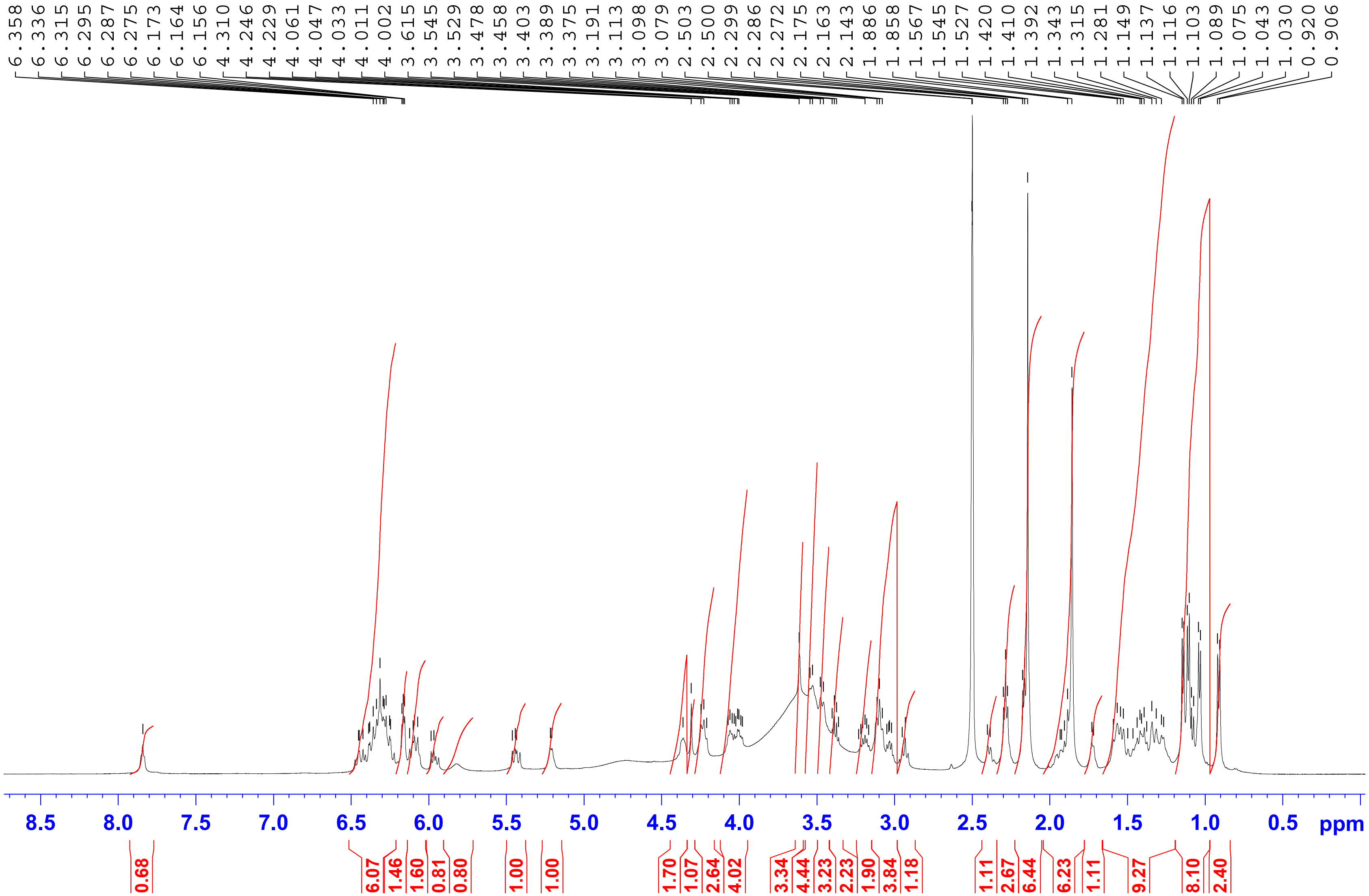

Figure S1. ^{1}H NMR spectra of the AmB derivative **1c.**

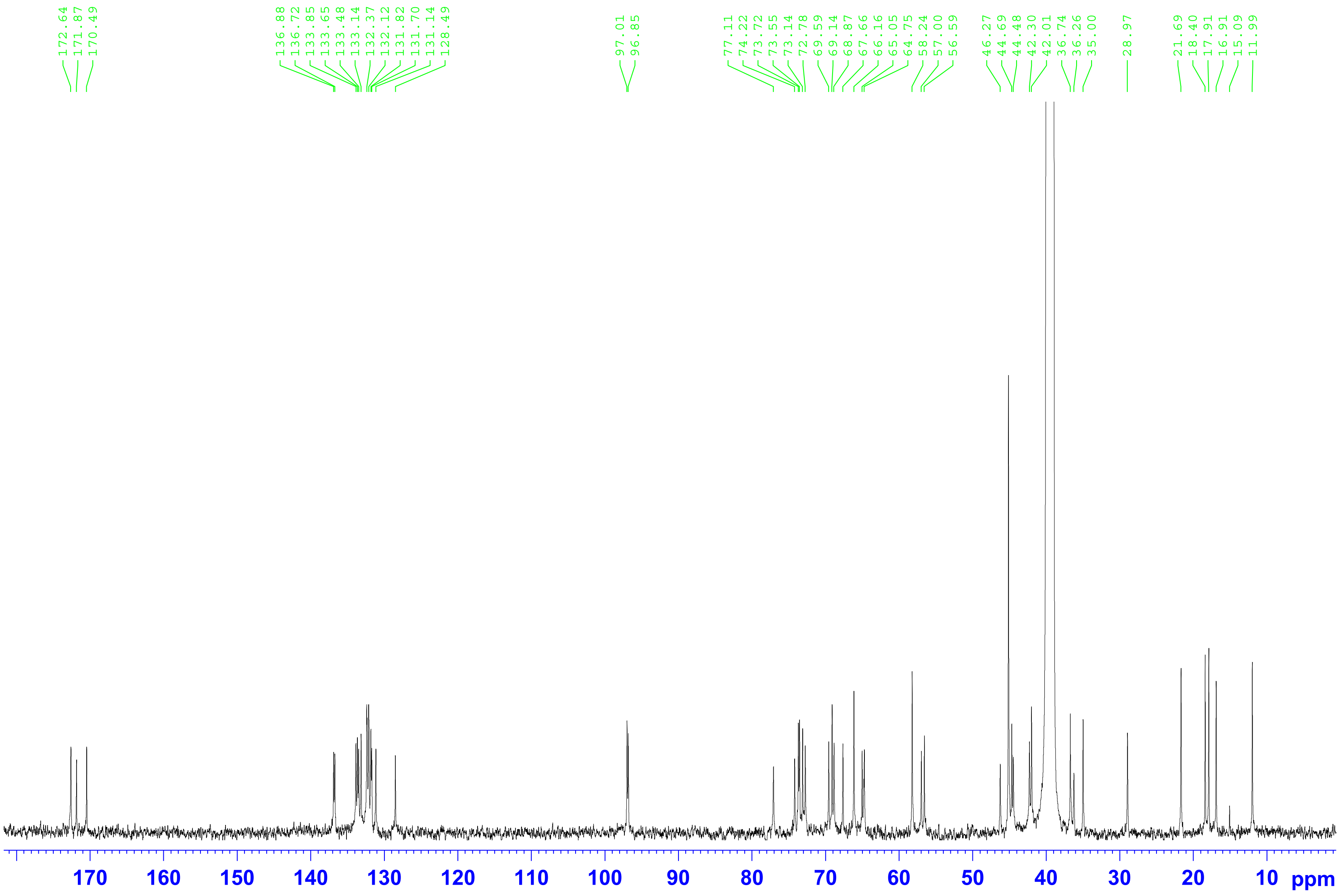

Figure S2. ^{13}C NMR spectra of the AmB derivative **1c.**

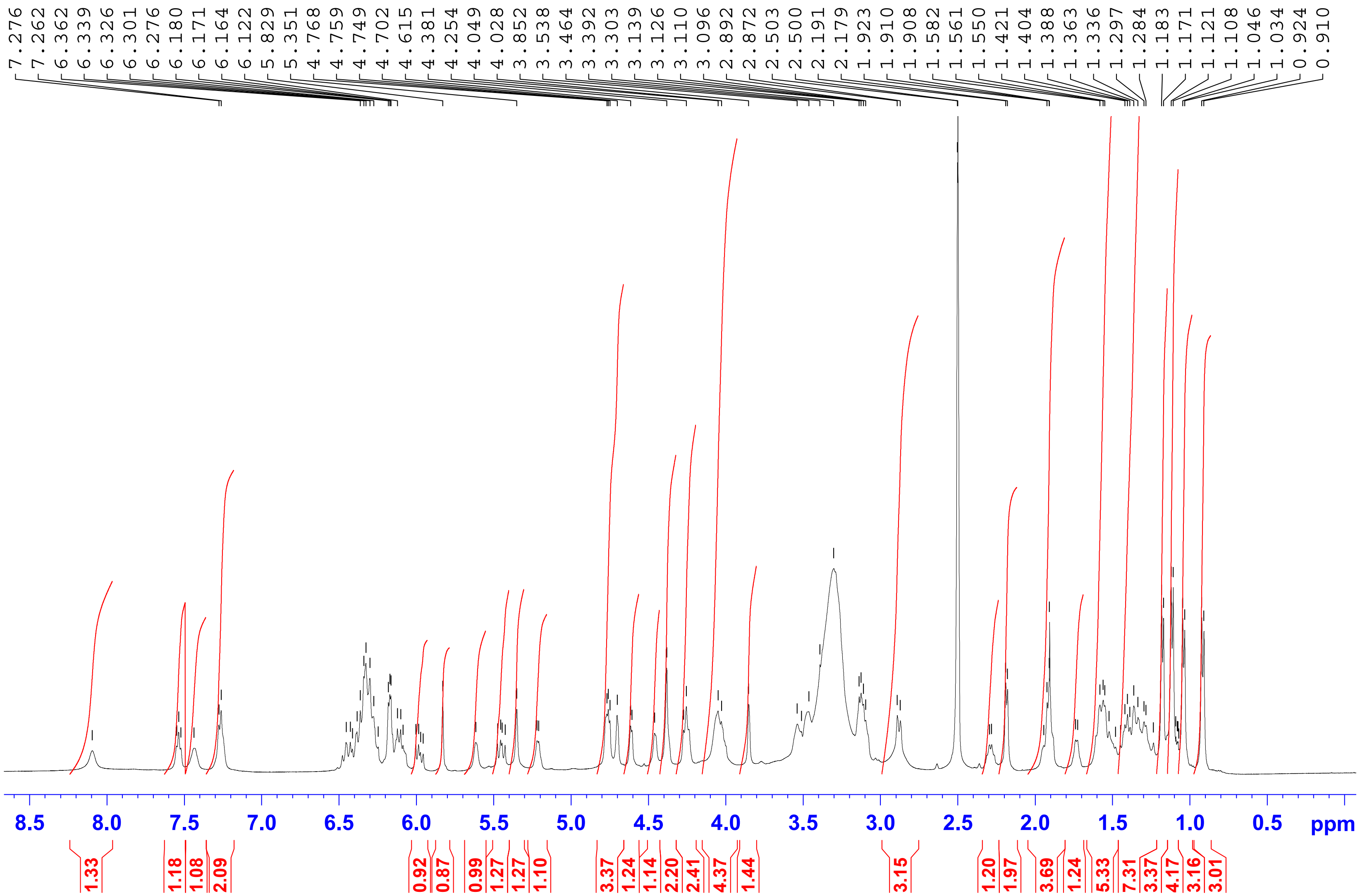

Figure S3. ^{1}H NMR spectra of the AmB derivative **1d.**

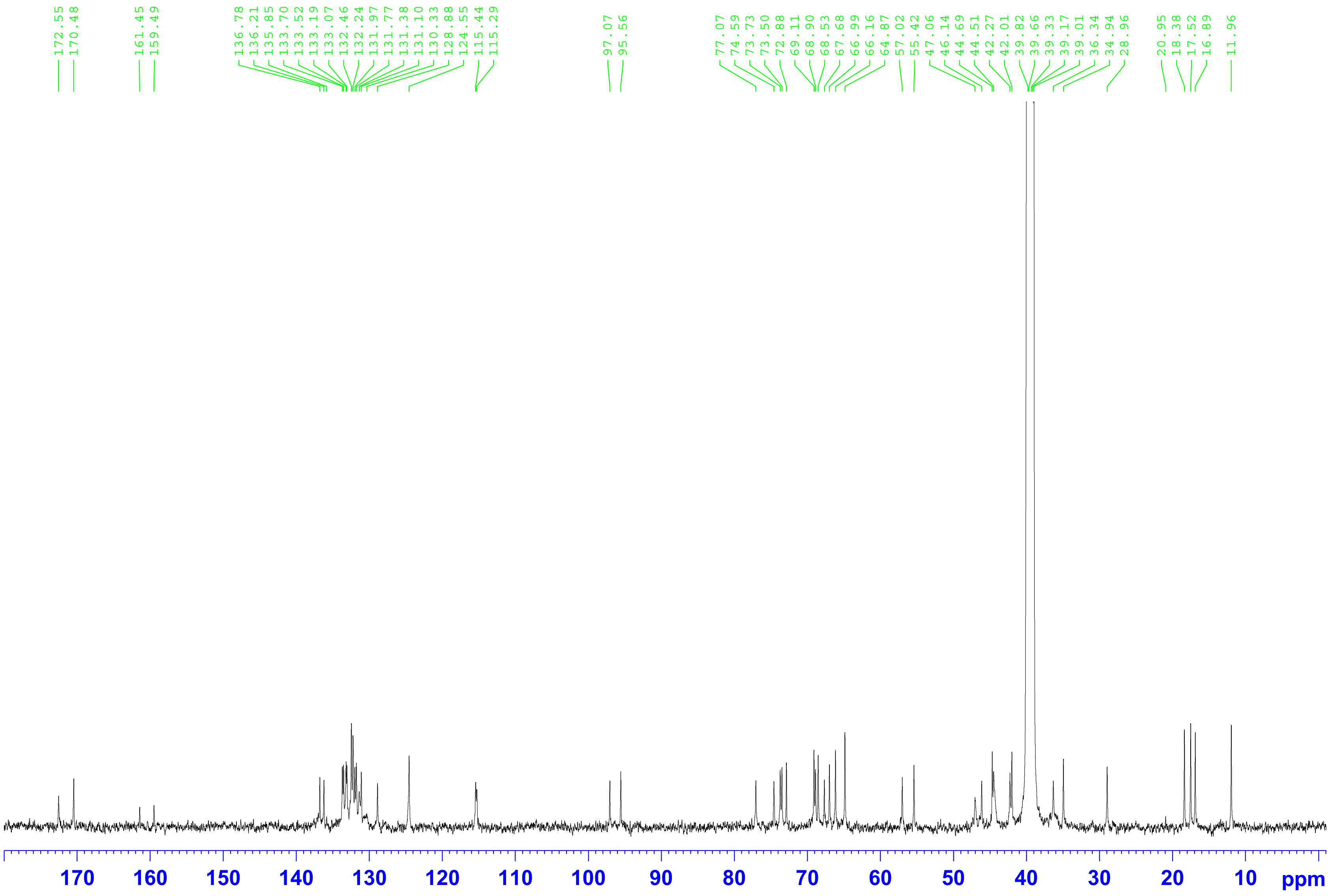

Figure S4. ^{13}C NMR spectra of the AmB derivative **1d.**

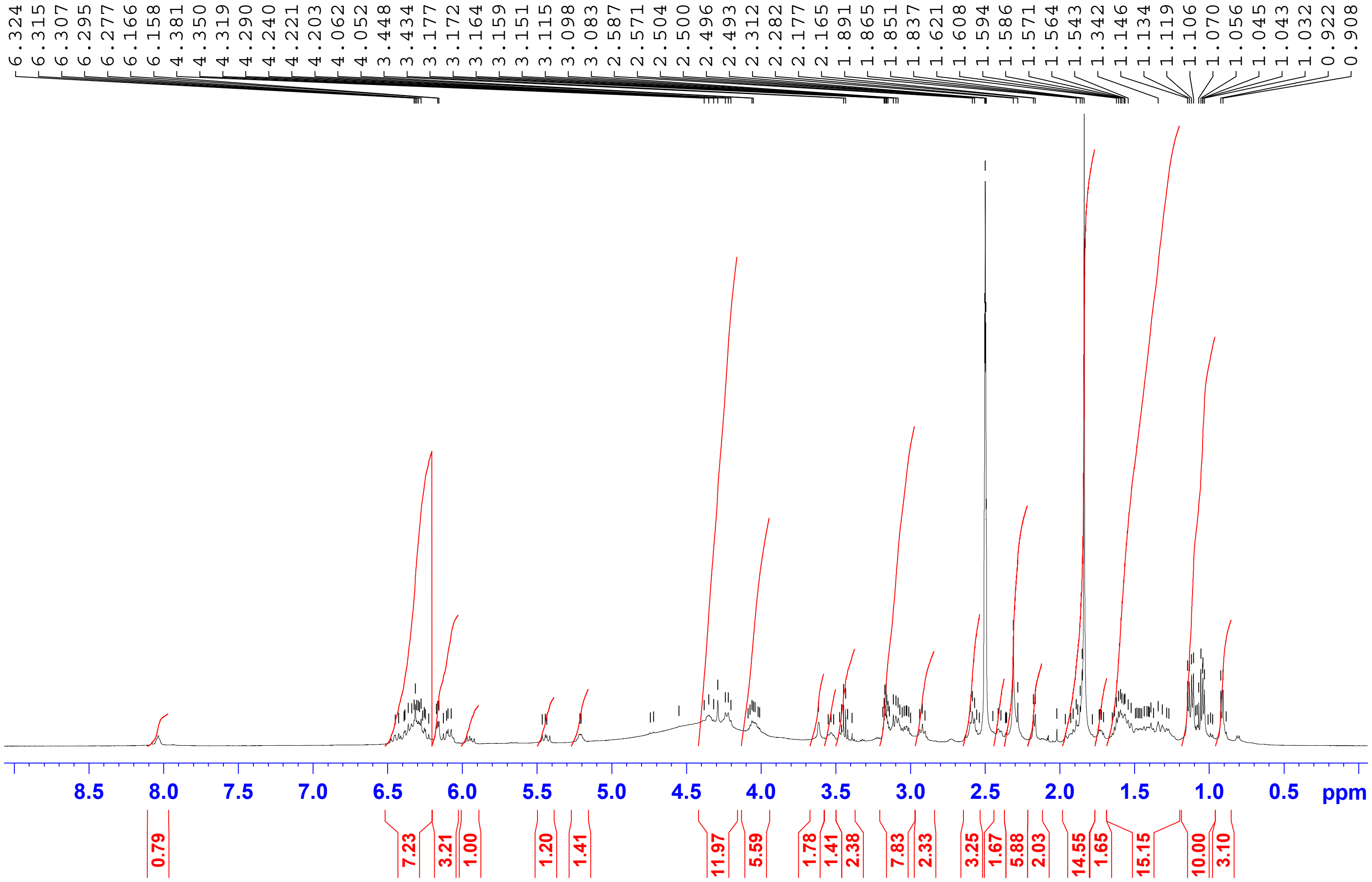

Figure S5. ^{1}H NMR spectra of the AmB derivative **1e.**

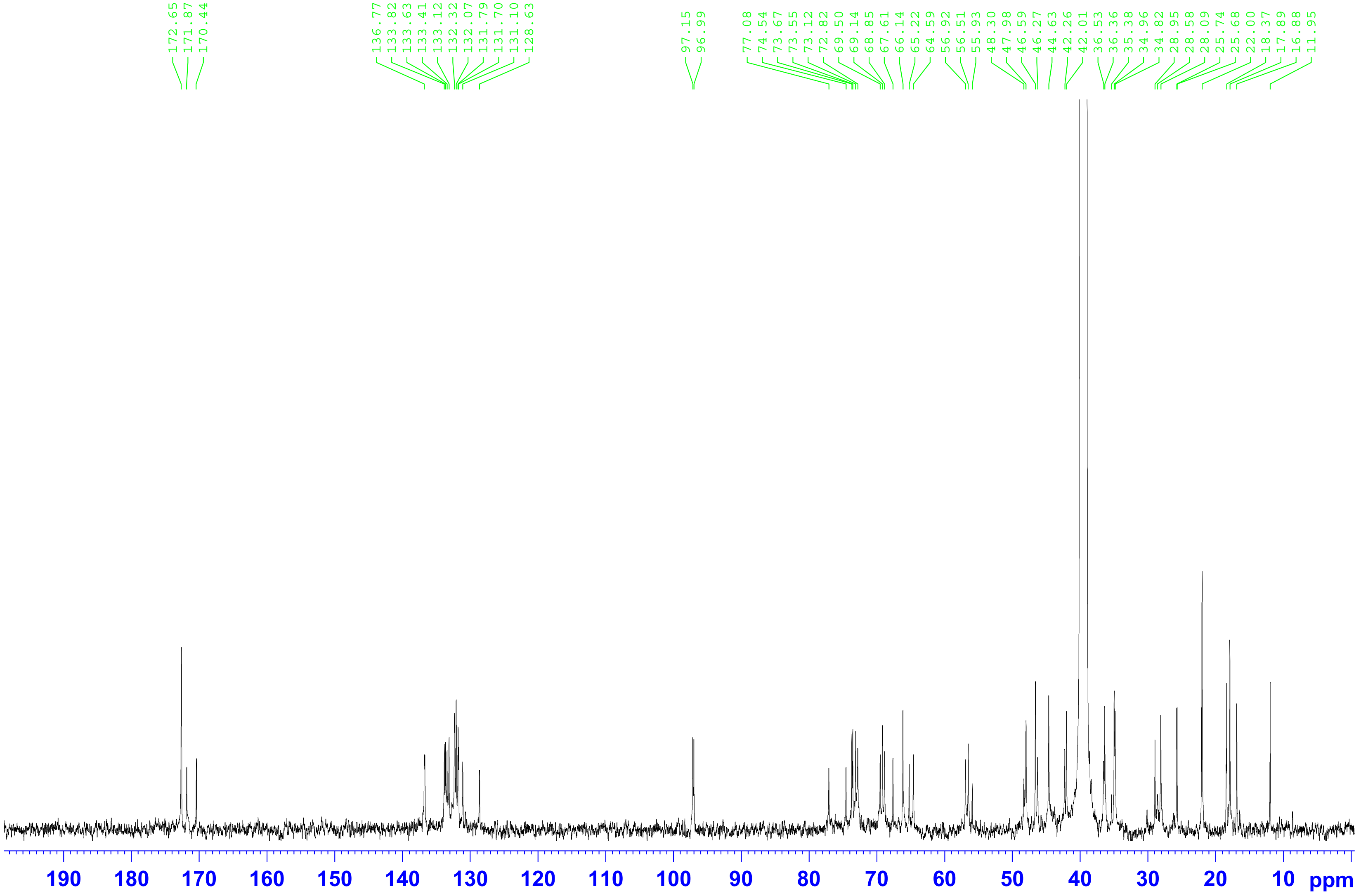

Figure S6. ^{13}C NMR spectra of the AmB derivative **1e**.

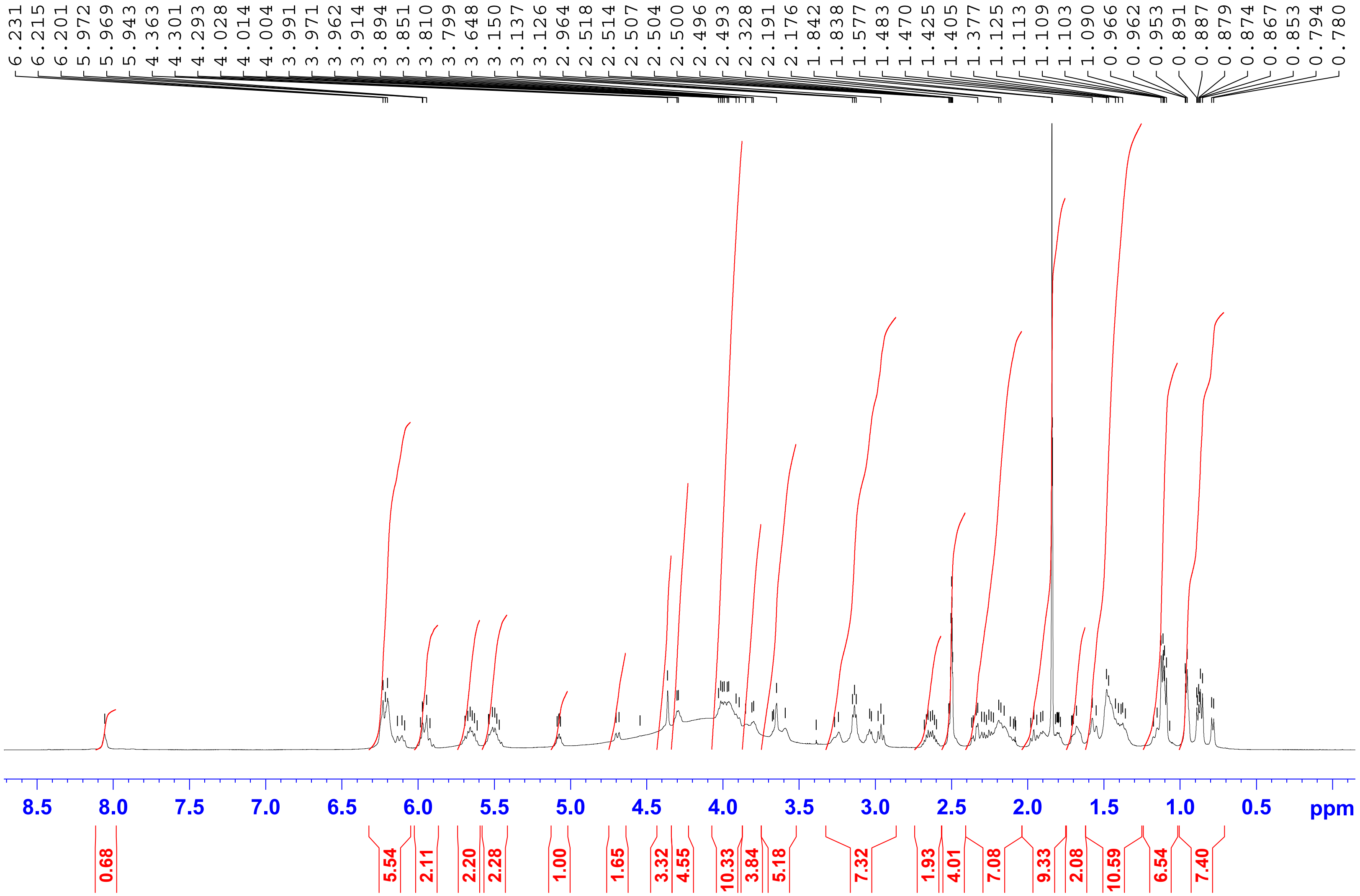

Figure S7. ^{1}H NMR spectra of the Nys derivative **2a**.

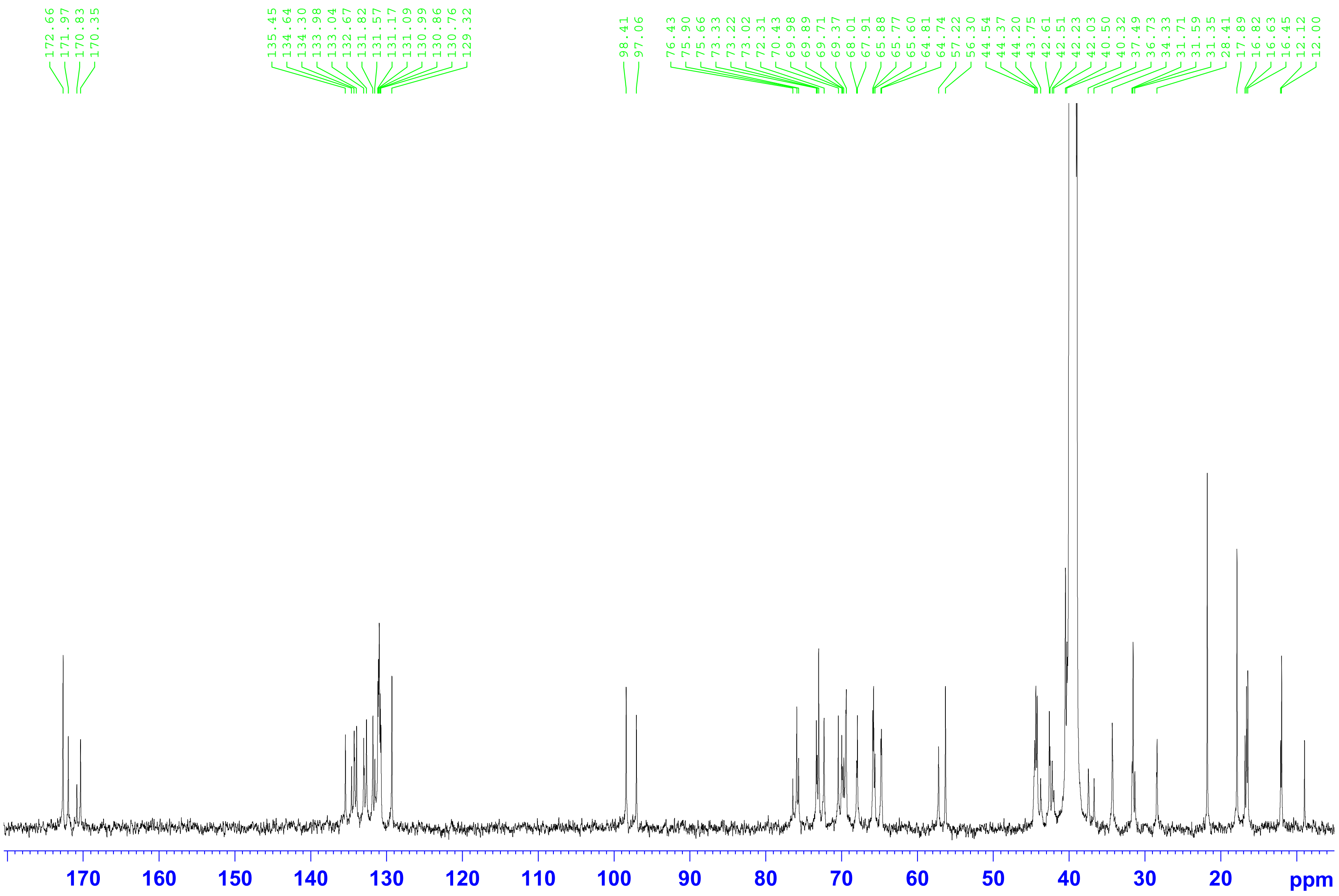

Figure S8. ^{13}C NMR spectra of the Nys derivative **2a**.

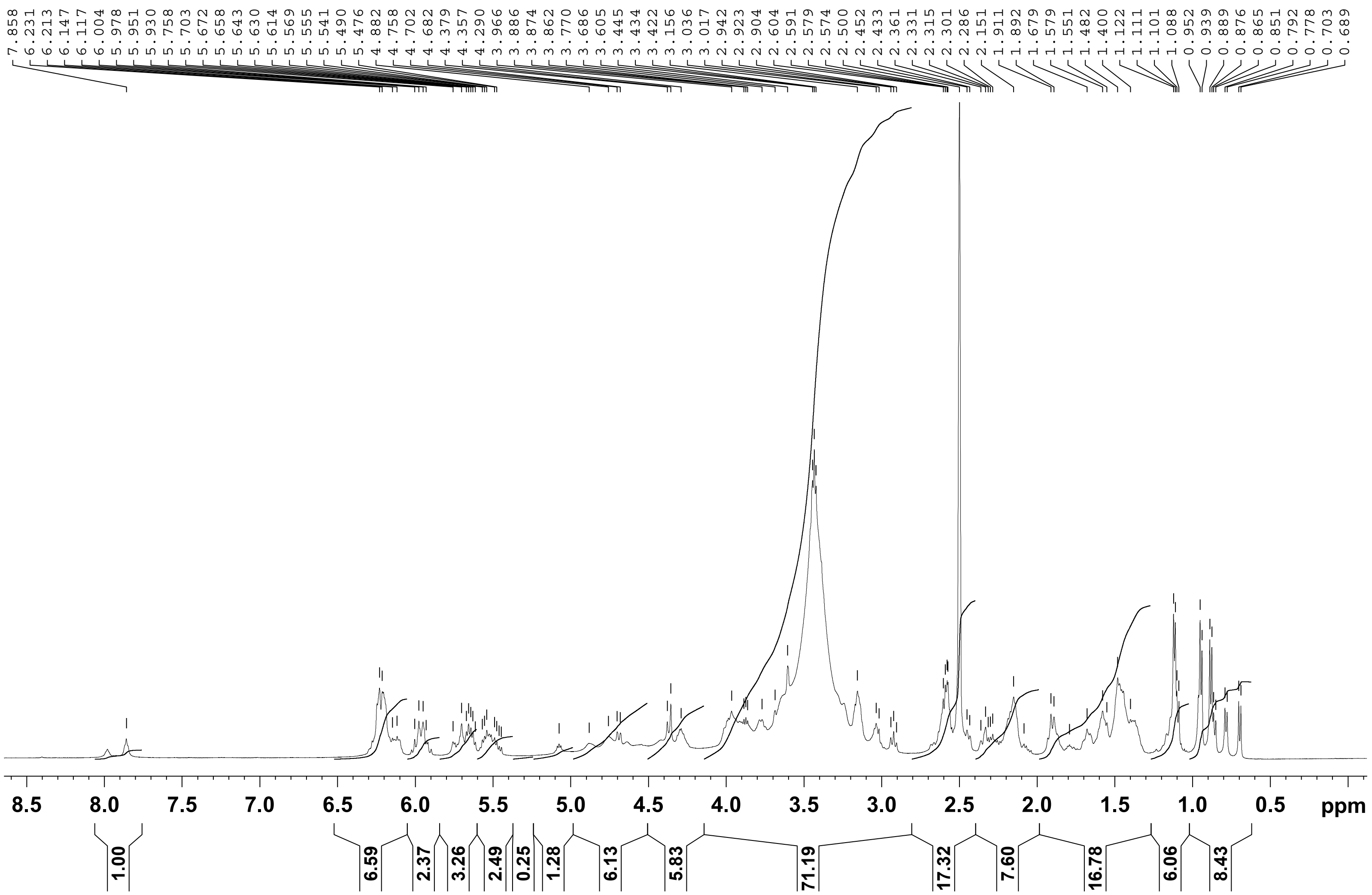

Figure S9. ^{1}H NMR spectra of the Nys derivative **2b**.

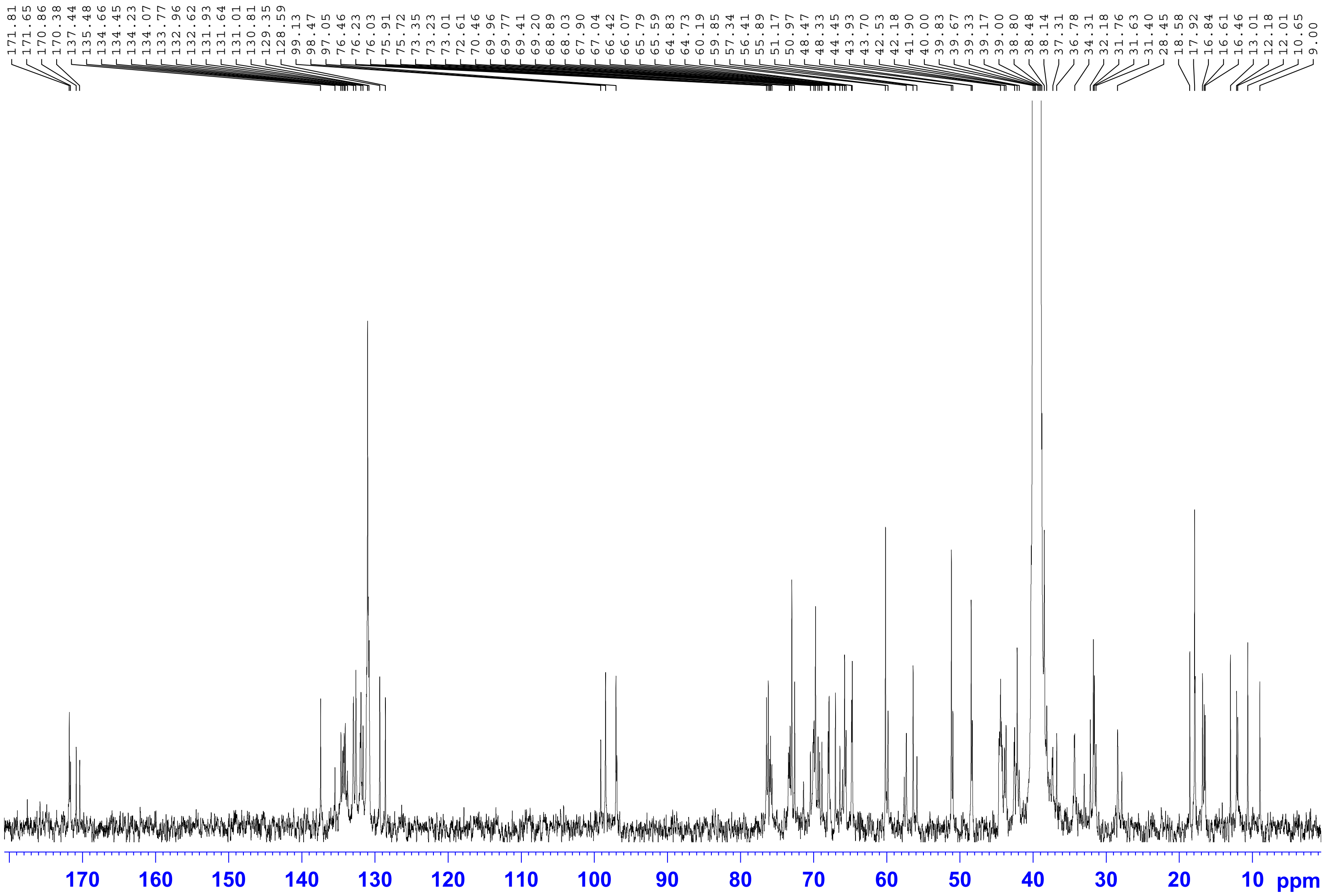

Figure S10. ^{13}C NMR spectra of the Nys derivative **2b**.

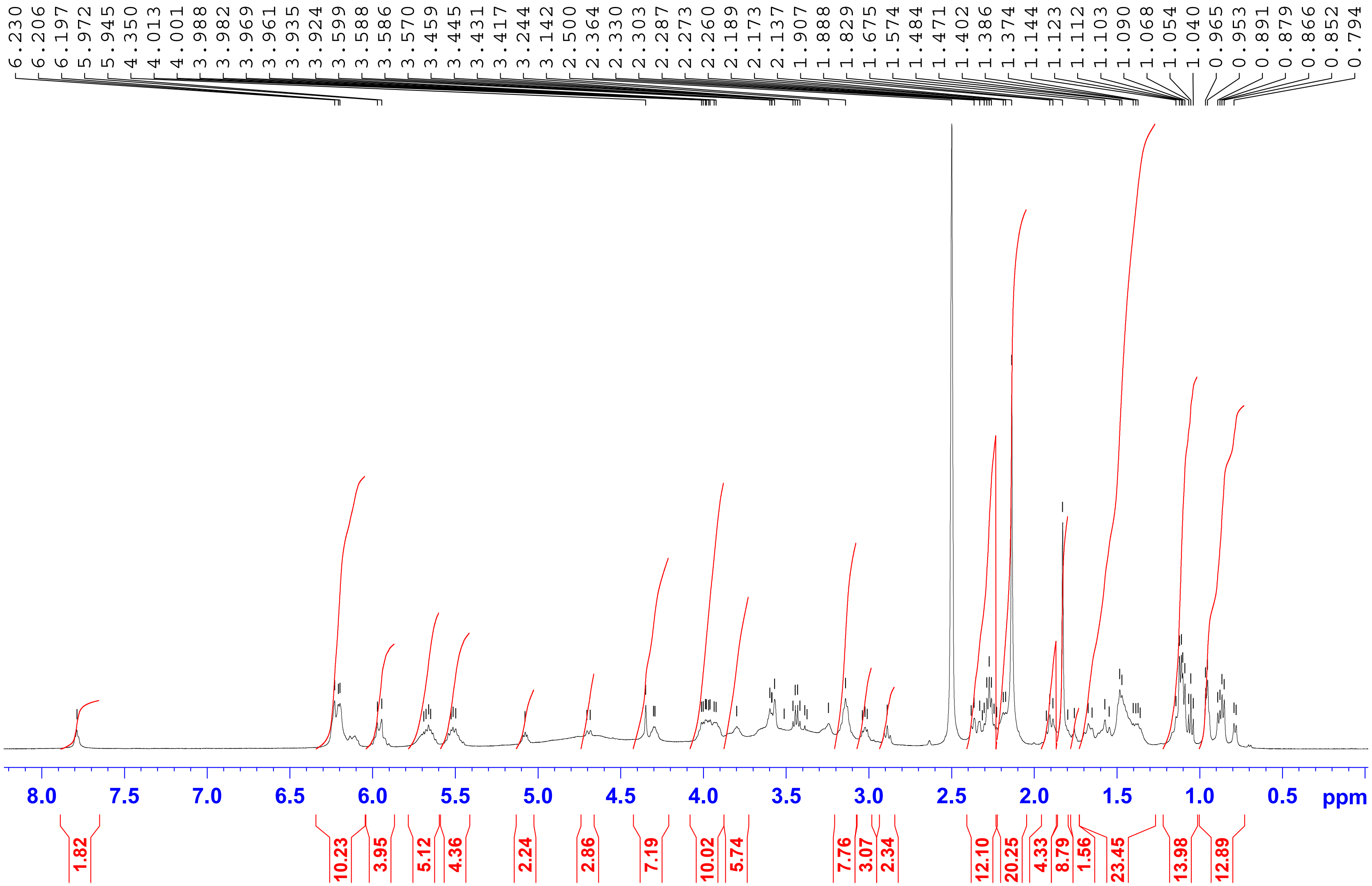

Figure S11. ^{1}H NMR spectra of the Nys derivative **2c**.

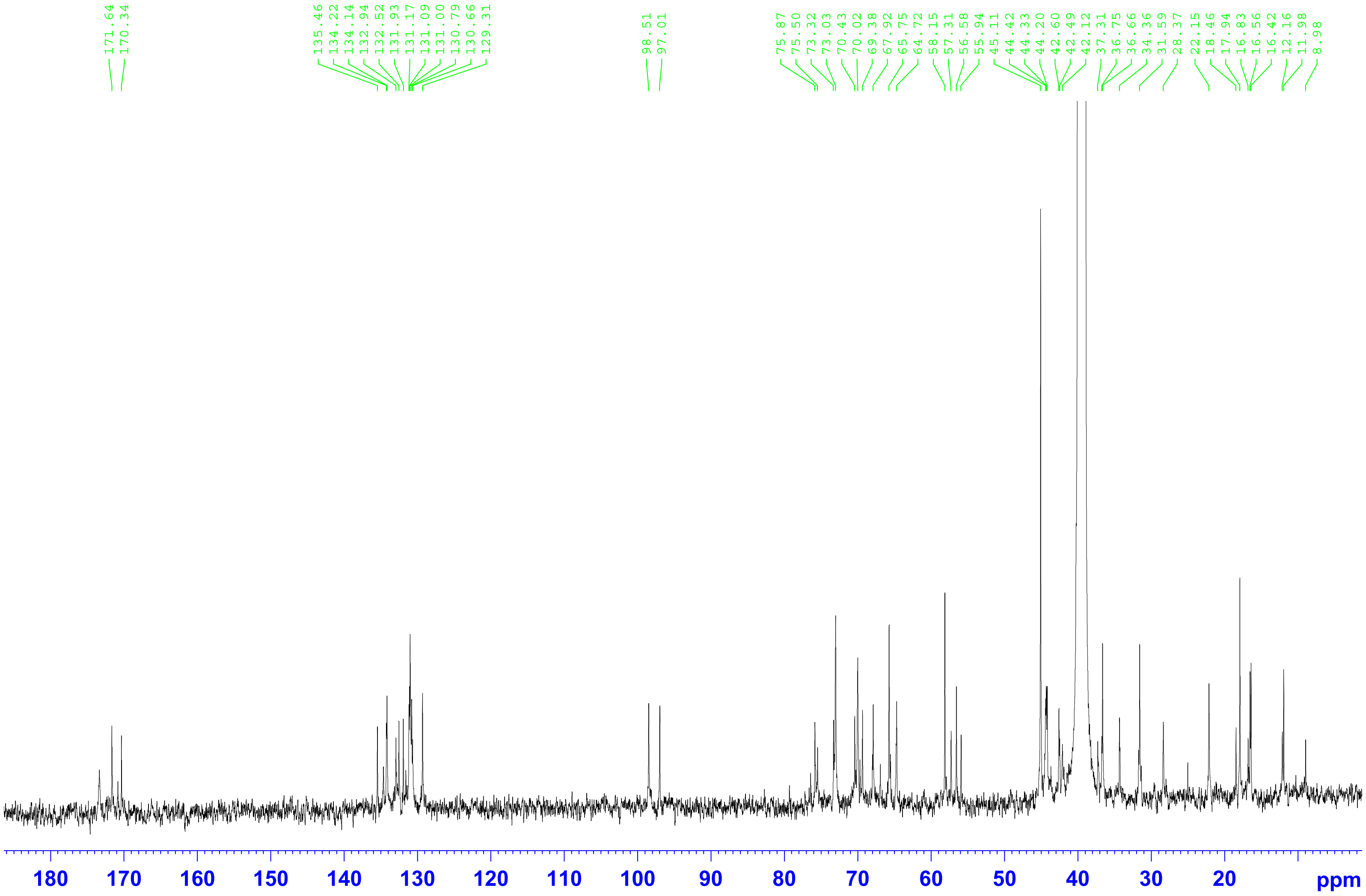

Figure S12. ^{13}C NMR spectra of the Nys derivative **2c**.

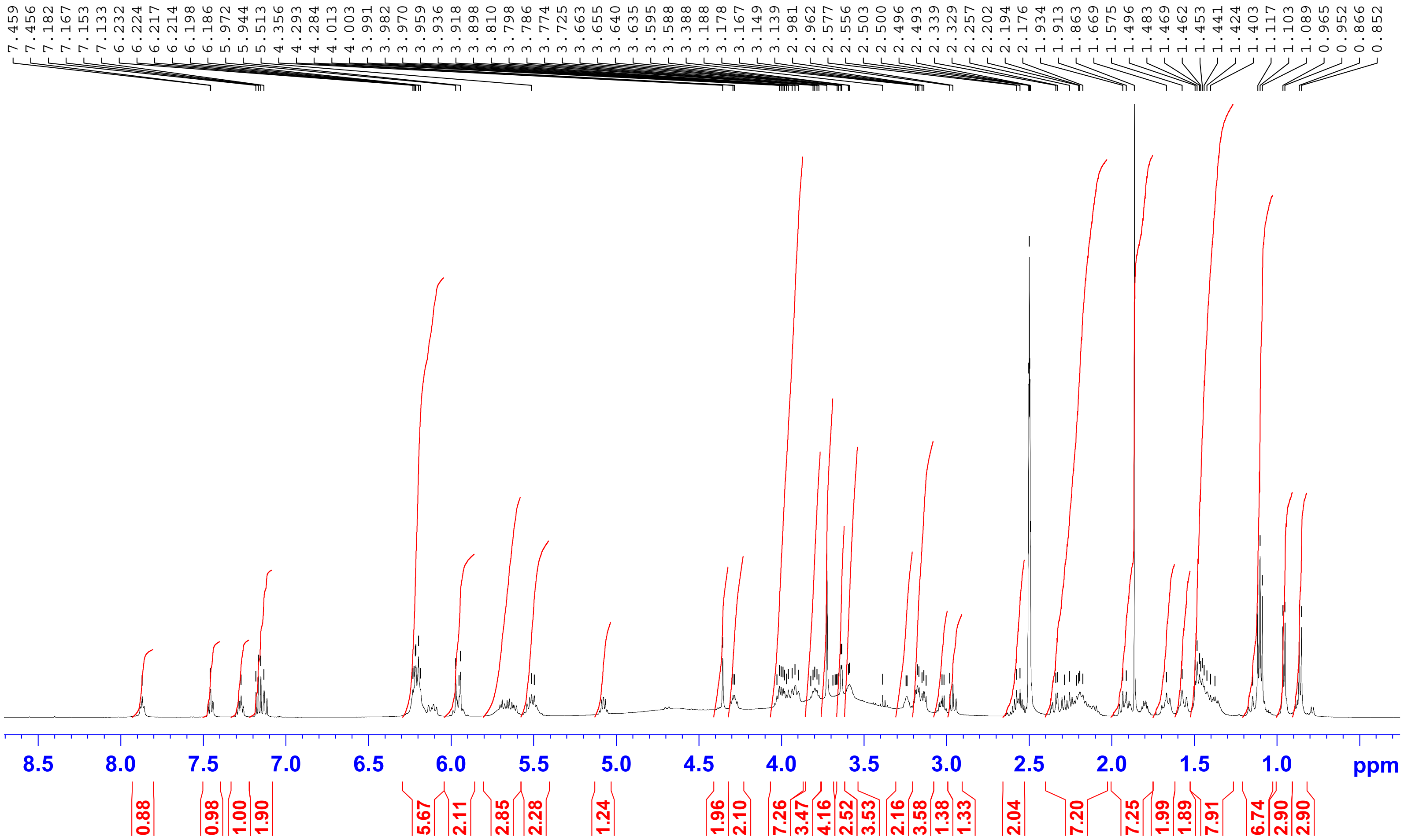

Figure S13. ^{1}H NMR spectra of the Nys derivative **2d**.

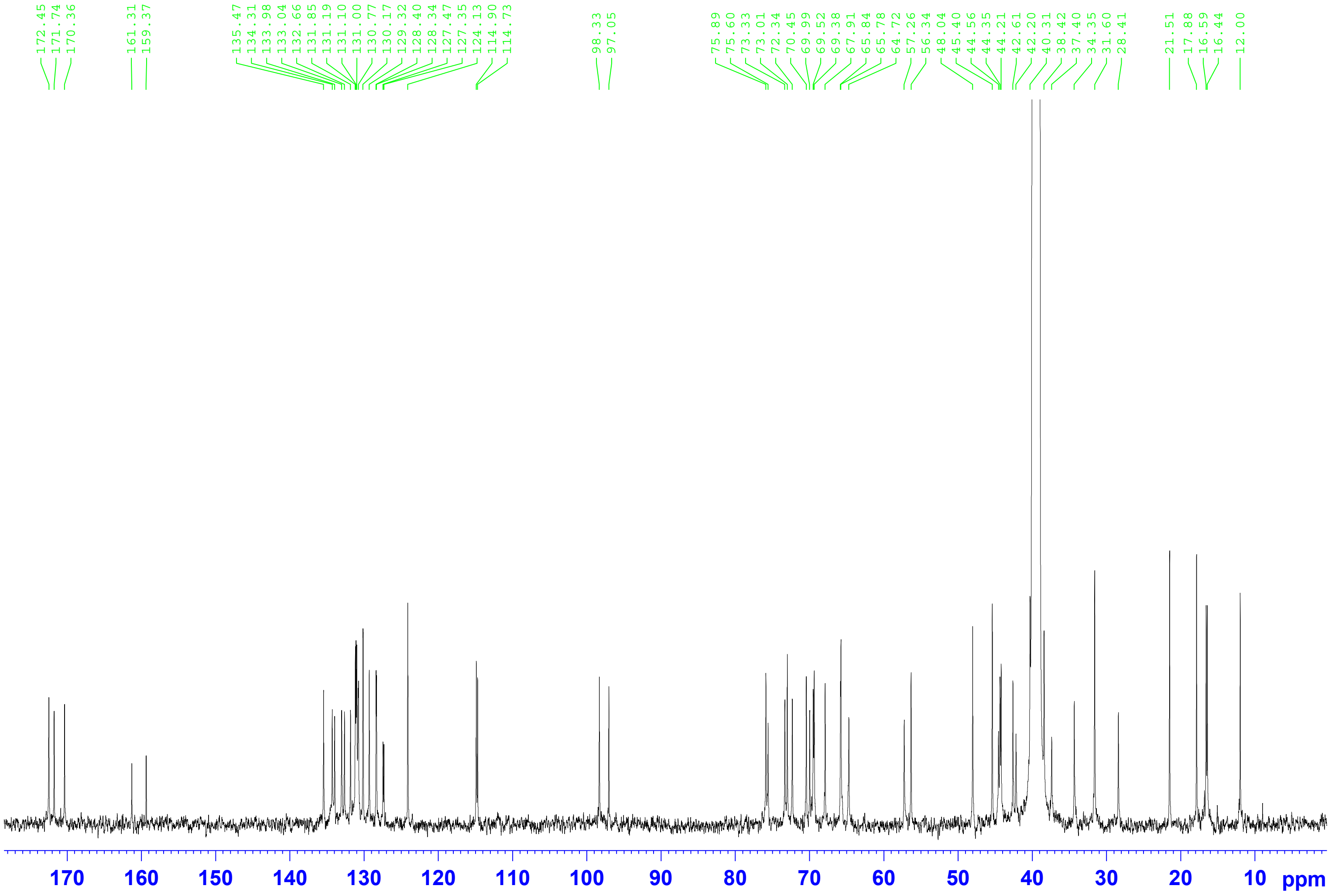

Figure S14. ^{13}C NMR spectra of the Nys derivative **2d.**

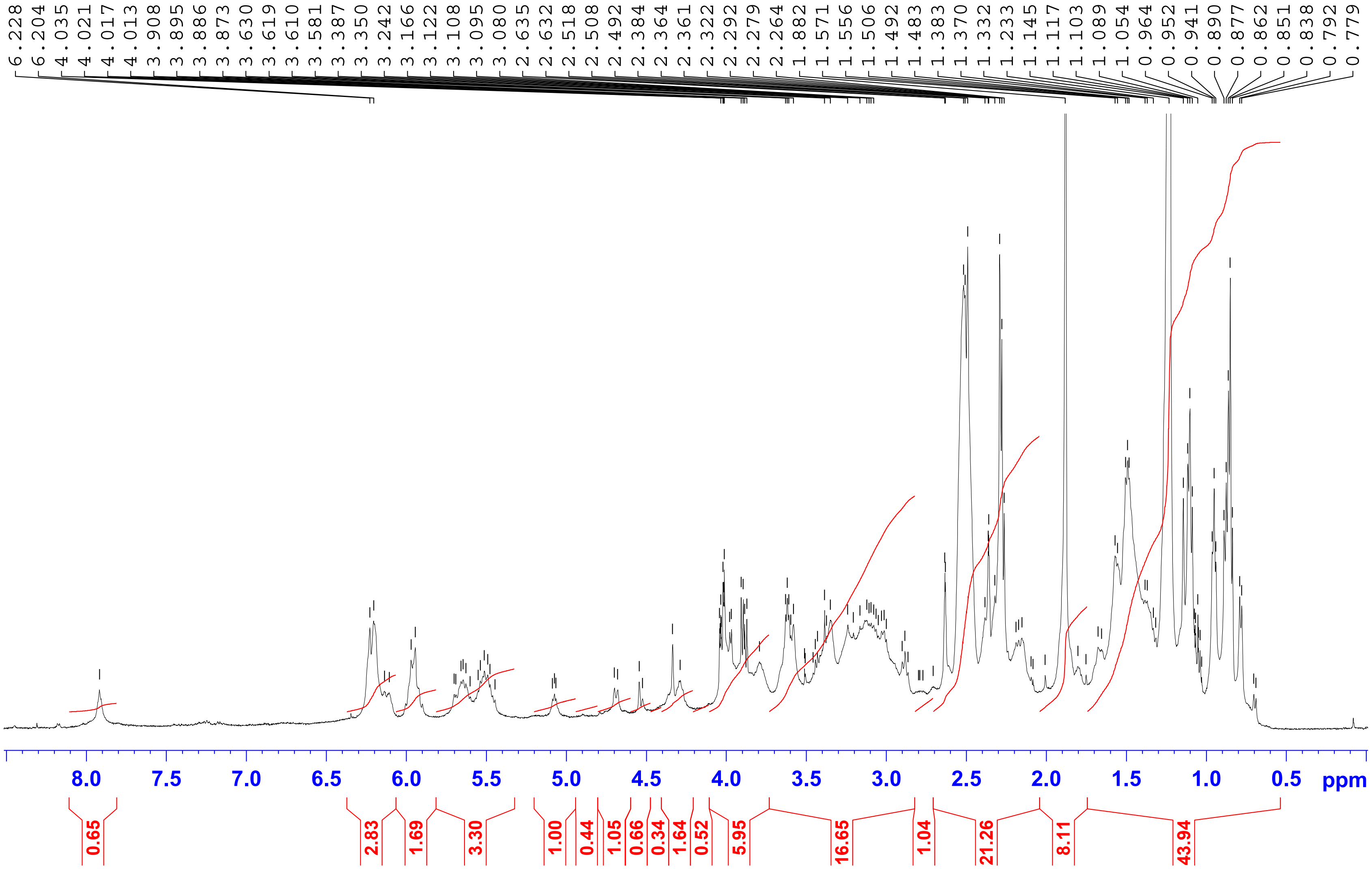

Figure S 15. ^{1}H NMR spectra of the **Nys** derivative **2e.**

.

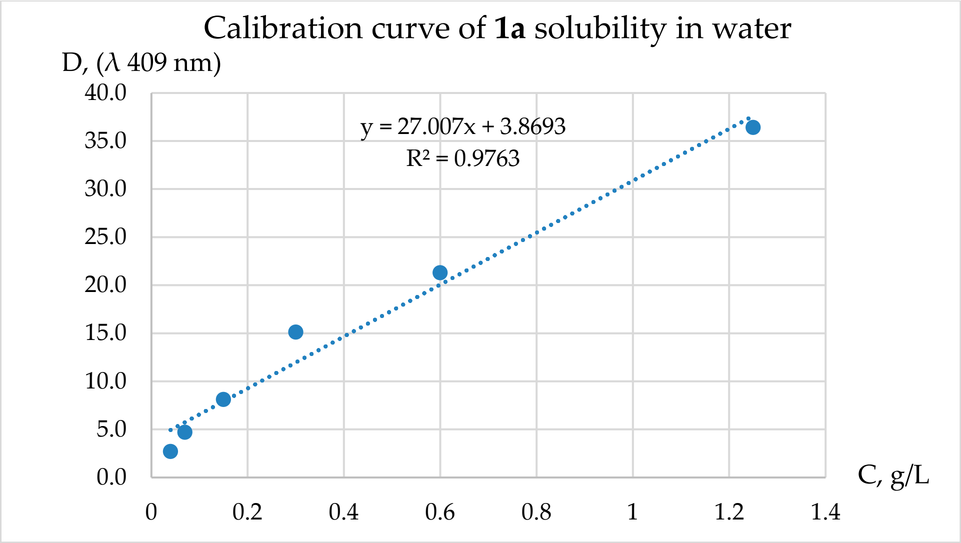

Figure S16. Calibration curve of the AmB derivative **1a**.

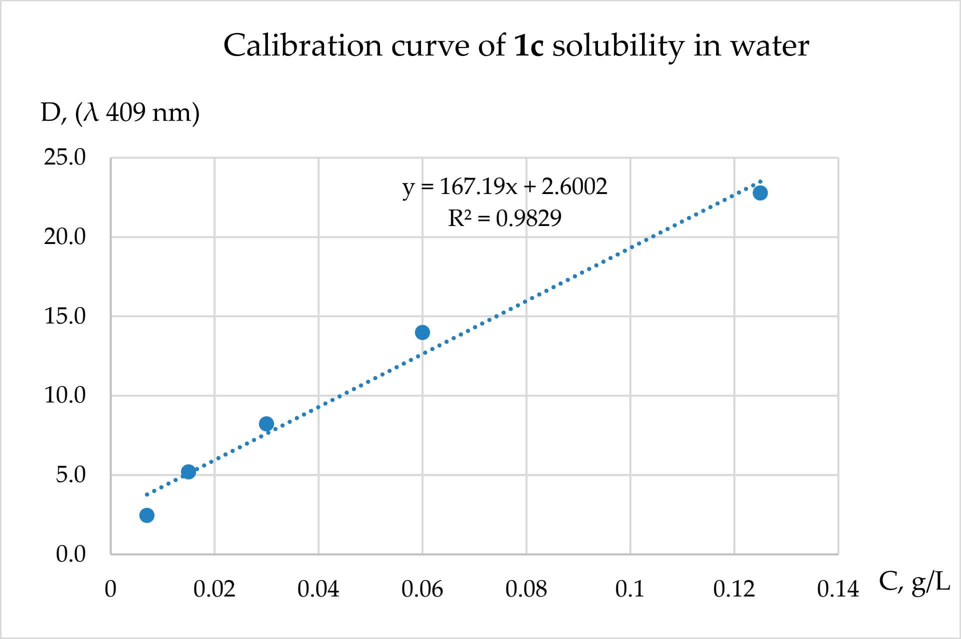

Figure S17. Calibration curve of the AmB derivative **1c**.

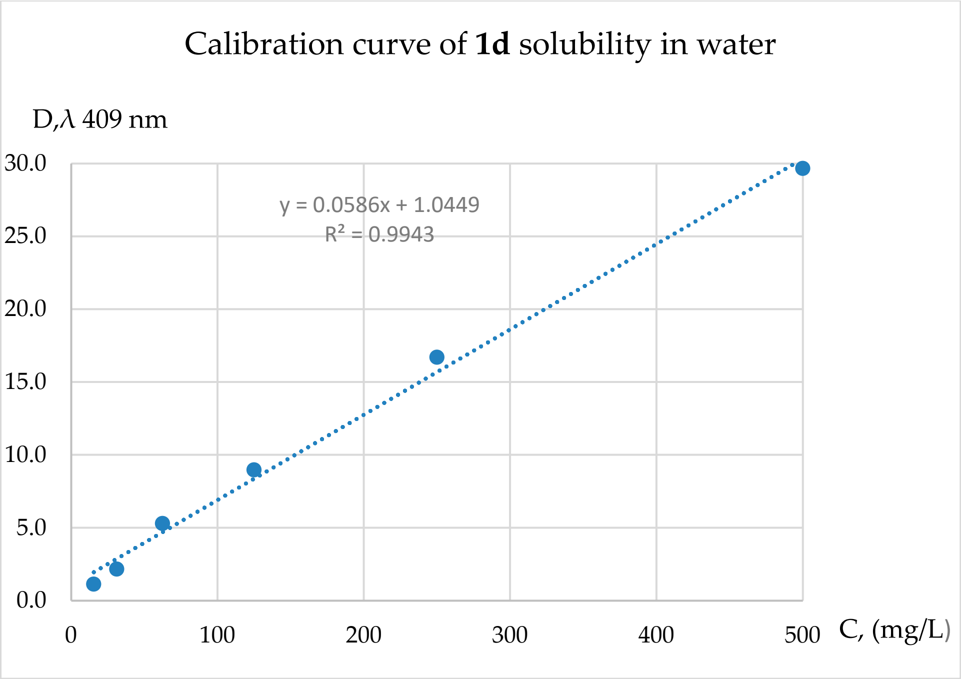

Figure S18. Calibration curve of the AmB derivative **1d**.

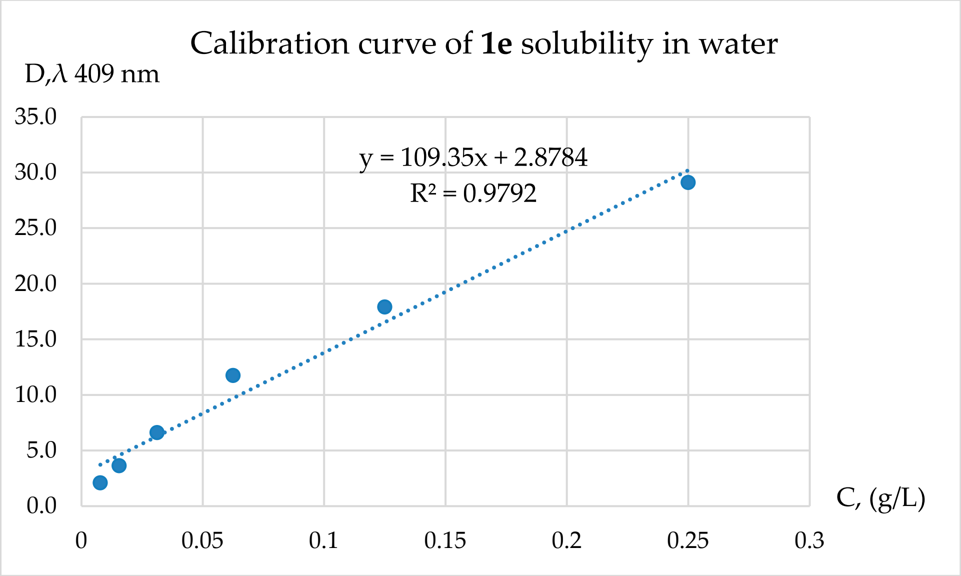

Figure S19. Calibration curve of the AmB derivative **1e**.

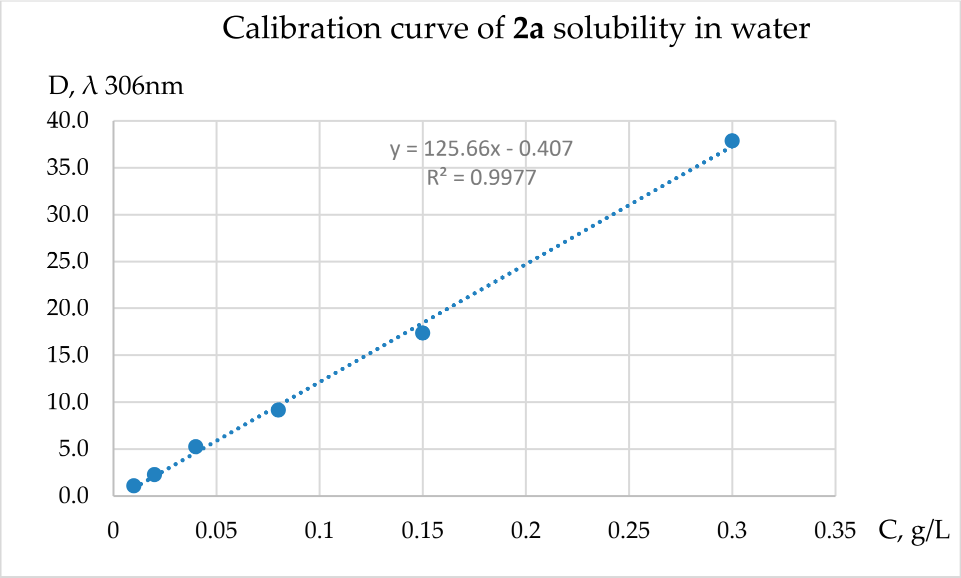

Figure S20. Calibration curve of the Nys derivative **2a**.

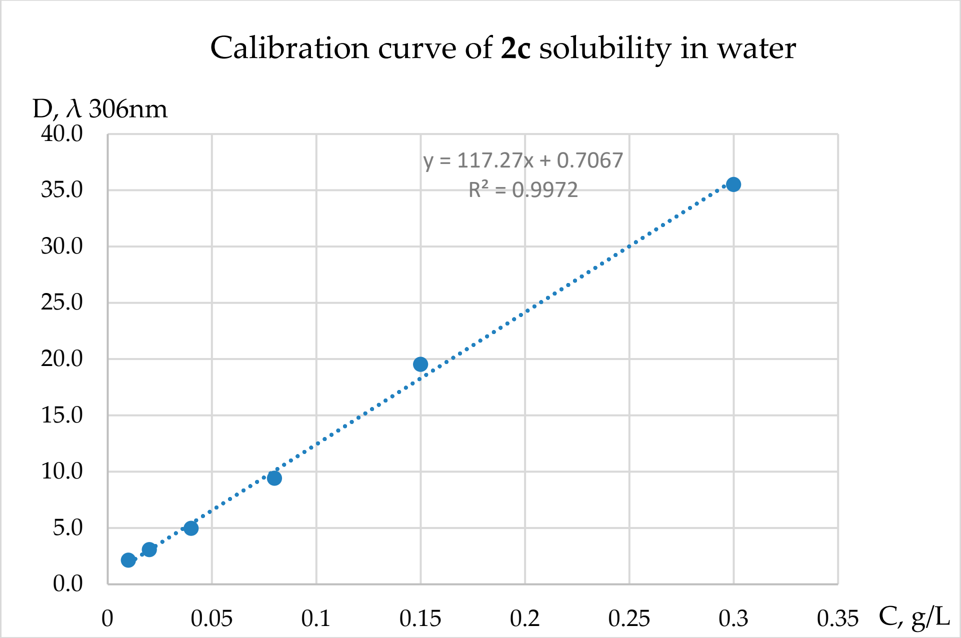

Figure S21. Calibration curve of the Nys derivative **2c**.

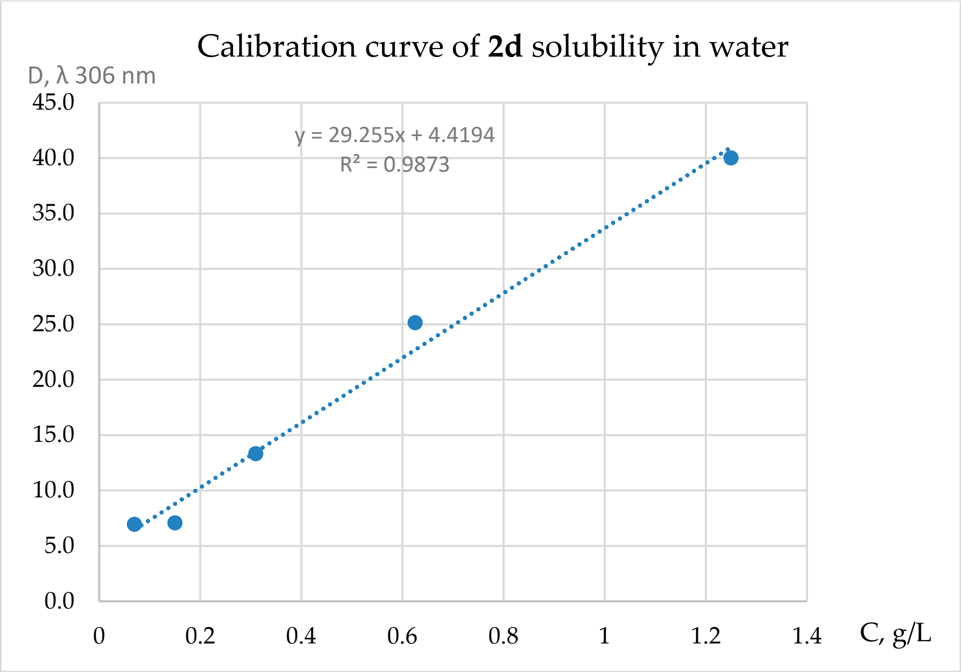

Figure S22. Calibration curve of the Nys derivative **2d**.

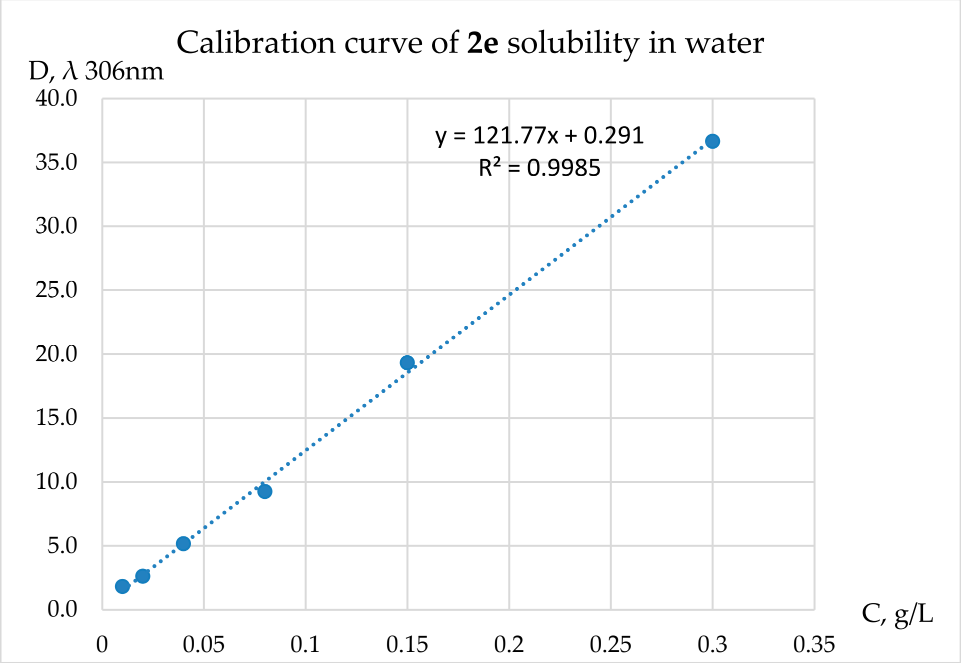

Figure S23. Calibration curve of the Nys derivative **2e**.
